# Whole genome sequencing of Ethiopian highlanders reveals conserved hypoxia tolerance genes

**DOI:** 10.1186/gb-2014-15-2-r36

**Published:** 2014-02-20

**Authors:** Nitin Udpa, Roy Ronen, Dan Zhou, Junbin Liang, Tsering Stobdan, Otto Appenzeller, Ye Yin, Yuanping Du, Lixia Guo, Rui Cao, Yu Wang, Xin Jin, Chen Huang, Wenlong Jia, Dandan Cao, Guangwu Guo, Victoria E Claydon, Roger Hainsworth, Jorge L Gamboa, Mehila Zibenigus, Guta Zenebe, Jin Xue, Siqi Liu, Kelly A Frazer, Yingrui Li, Vineet Bafna, Gabriel G Haddad

**Affiliations:** 1 Bioinformatics & Systems Biology Graduate Program, University of California San Diego, La Jolla, California 92093, USA; 2 Department of Pediatrics, Division of Respiratory Medicine, University of California San Diego, La Jolla, California 92093, USA; 3 BGI-Americas, Cambridge, Massachusetts 02142, USA; 4 Department of Neurology, New Mexico Health Enhancement and Marathon Clinics Research Foundation, Albuquerque, New Mexico 87122, USA; 5 BGI-Shenzhen, Shenzhen 518083, China; 6 Department of Biomedical Physiology and Kinesiology, Faculty of Science, Simon Fraser University, British Columbia, Canada V5A 16S; 7 Division of Cardiovascular and Neuronal Remodeling, Faculty of Medicine, University of Leeds, Leeds LS2 9JT, UK; 8 Division of Clinical Pharmacology/Department of Medicine, Vanderbilt University Medical Center, Tennessee 37232, USA; 9 Department of Medicine, Yehuleshet Higher clinic, University of Addis Ababa, Addis Ababa 1176, Ethiopia; 10 Beijing Institute of Genomics, Chinese Academy of Sciences, Beijing 100029, China; 11 Department of Pediatrics, Division of Genome Information Science, University of California San Diego, La Jolla, California 92093, USA; 12 Department of Computer Science and Engineering, University of California San Diego, La Jolla, California 92093, USA; 13 Department of Neurosciences, University of California San Diego, La Jolla, California 92093, USA; 14 Rady Children’s Hospital, San Diego, California 92123, USA

## Abstract

**Background:**

Although it has long been proposed that genetic factors contribute to adaptation to high altitude, such factors remain largely unverified. Recent advances in high-throughput sequencing have made it feasible to analyze genome-wide patterns of genetic variation in human populations. Since traditionally such studies surveyed only a small fraction of the genome, interpretation of the results was limited.

**Results:**

We report here the results of the first whole genome resequencing-based analysis identifying genes that likely modulate high altitude adaptation in native Ethiopians residing at 3,500 m above sea level on Bale Plateau or Chennek field in Ethiopia. Using cross-population tests of selection, we identify regions with a significant loss of diversity, indicative of a selective sweep. We focus on a 208 kbp gene-rich region on chromosome 19, which is significant in both of the Ethiopian subpopulations sampled. This region contains eight protein-coding genes and spans 135 SNPs. To elucidate its potential role in hypoxia tolerance, we experimentally tested whether individual genes from the region affect hypoxia tolerance in *Drosophila*. Three genes significantly impact survival rates in low oxygen: *cic*, an ortholog of human *CIC*, *Hsl*, an ortholog of human *LIPE*, and *Paf-AHα*, an ortholog of human *PAFAH1B3*.

**Conclusions:**

Our study reveals evolutionarily conserved genes that modulate hypoxia tolerance. In addition, we show that many of our results would likely be unattainable using data from exome sequencing or microarray studies. This highlights the importance of whole genome sequencing for investigating adaptation by natural selection.

## Background

Humans have occupied high altitude regions for thousands of years [1]. It is estimated that currently more than 140 million people live and work at altitudes above 2,500 m [2], where hypoxic conditions present a challenge for survival. Previous studies suggest that the three large high altitude populations (that is, Andeans, Himalayans, and Ethiopians) have each adapted uniquely to cope with their inhospitable hypoxic environments [3,4]. It has also been suggested that the Ethiopians are better adapted to these conditions, as they show the least evidence of chronic mountain sickness (CMS), a high altitude syndrome that exists in other populations, especially the Andeans [5]. For instance, of the three highlander populations, Ethiopians show arterial oxygen levels that are most similar to sea level controls [4,6]. Although it has long been proposed that genetic factors contribute to adaptation to high altitude, these remain largely unproven [7]. Recent advances in high-throughput sequencing technologies have made it feasible to analyze patterns of genetic variation in human populations across the entire genome. To date, several genomic scans for natural selection have been performed in high altitude populations (for instance, [8-15]); however, as these studies either focused on *a priori* candidate genes, or assayed small portions of the genome (exons or a subset of genotyped SNPs), there is likely much yet to be deciphered.

We previously performed the first whole genome resequencing-based analysis of genes contributing to hypoxia adaptation in a tolerant *Drosophila* strain, generated through laboratory evolution [16]. Examining the complete genome of adapted populations, we were able to detect fine changes in the allele frequency spectrum consistent with natural selection. Extending the analytical strategy from our previous study, we present here the results of a whole genome resequencing-based analysis identifying genes that likely contribute to high altitude adaptation in humans. We focused our study on 13 high altitude (approximately 3,500 m) native Ethiopian residents. Specifically, we analyzed the genomes of six individuals of Oromo heritage living on Bale Plateau (labeled 'Oromos'), and seven individuals residing on the Chennek field in the Simien Mountains (labeled 'Amhara').

While our study uses well-known statistical tests to identify genomic regions undergoing a selective sweep, we have made a number of novel design choices compared with previous studies of human adaptation [8-15]. First, we use whole genome sequencing (WGS) rather than genotyping arrays, allowing a much richer sampling of the site frequency spectrum in a given region. Second, in order to test whether particular genes highlighted by our analysis play a role in hypoxia tolerance, we used RNA interference (RNAi) to target their respective orthologs in *Drosophila melanogaster*. Thus, in addition to reporting a set of regions showing a strong signature of positive selection in high altitude populations (as in previous studies), we also report on several genes that, apart from showing such a signature, are shown experimentally to modulate the adaptive phenotype.

## Results

We sequenced the genome of each individual using Illumina’s Hiseq 2000 platform to a mean genome-wide depth of approximately 18× per individual. We mapped the reads to the hg19 human reference using BWA [17], and performed variant calling using the GATK pipeline [18,19]. See Additional file 1 for an overview of the computational pipeline.

We then used ADMIXTURE [20] to identify the closest populations from the 1000 Genomes Project [21], release 20100804. This showed that our Ethiopian highlanders share common genetic ancestry, and are largely an admixture of two ancestral groups (Additional file 2). The largest ancestry component shows high similarity to African populations, particularly the Luhya (LWK), located in neighboring Kenya. The remainder is largely shared with individuals of non-Finnish, European ancestry. As a result, for lowlander controls, we used variant calls from low coverage whole-genome sequencing of 67 Luhya (LWK) individuals. As an out-group we used 90 northern European ancestry (CEU) individuals. We also performed principle component analysis (Additional file 3) on our study populations jointly with the lowlander controls (LWK) and out-group (CEU), further illustrating our study populations as an admixture of these two ancestral groups. Due to differences in coverage between the control populations and our Ethiopian sequence data, we filtered low coverage or poor quality loci prior to testing for selection (see Materials and methods).

### Genome-wide scans of selection

Under environmental selective stress, such as hypoxia, alleles that confer an adaptive advantage are likely to increase in frequency, along with their linked neighbors. This process is known as a 'selective sweep'. We sought regions with evidence of such a sweep: a loss of genetic diversity in the region and a corresponding decrease in the scaled mutation rate, *θ* (=4*N*_*e*_*μ* where *N*_*e*_ is the effective population size and μ is the mutation rate). We computed four cross-population test statistics (denoted S_f_, S_π_, F_st_, and population branch statistic (PBS); see Materials and methods) that measure this loss in diversity. Cross-population tests provide a control for locus-specific variability in scaled mutation rates, enabling a direct comparison of the effective population size as a measure of selection (S_f_ and S_π_). They also allow for an estimation of branch lengths and bottlenecks relative to the point of divergence between populations (F_st_ and PBS). Through extensive simulations, we showed that the power of these tests varies depending on the selection coefficient and time since selection, among other things [22] (Materials and methods; Additional files 4 and 5). As these parameters are unknown, we considered regions that were significant under any of these tests.

We assume that the genetic basis for the adaptation to low oxygen influences relatively few loci genome-wide. As a result, for a cross-population test, the null distribution of two neutrally evolving populations can be approximated by the observed distribution of highlanders versus lowlander controls. We report regions exceeding the top 0.1% genome-wide value for each test. For the Amhara population, these values were 0.16 (PBS), 0.18 (F_st_), 1.73 (S_π_), and 2.0 (S_f_). For the Oromos, these were 0.15 (PBS), 0.16 (F_st_), 1.61 (S_π_), and 1.88 (S_f_).

We initially identified 420 regions spanning 36.8 Mbp as significant in at least one test under the corresponding 0.1% genome-wide false discovery rate (FDR; see Additional file 6 for a summary, and Additional file 7 for the complete set of regions identified by the four tests). While genome-wide scans for selection are a powerful tool for detecting genetic factors contributing to adaptation, it is also true that these scans make no guarantees on the significance of the results [23,24]. Consequently, we provide experimental evidence to further support the role of some of our highlighted genes in hypoxia adaptation. Due to the infeasibility of doing this for all genes identified by our tests, we were faced with the need to prioritize candidate regions that showed the strongest evidence of testable selection, and that appeared unique to Ethiopian highlanders. We thus implemented a series of automated prioritization criteria.

### Region prioritization

#### Frequency block differential relative to lowlander control

A region under strong positive selection is characterized by changes in allele frequencies that cannot be explained by a neutral model. These often manifest as blocks of SNPs with increased minor allele frequency. We leverage this fact by seeking regions with multiple SNPs present in a block structure, at comparatively high frequencies in the highlander populations. Given a population sample of size *n*, we iterate over all possible frequency values *f*, where *f* = (1/*n*, 2/*n*, …, (*n*-1)/*n*). For each value of *f*, we isolate the variants in the region of frequency within 1/*n* from *f*. From these, we define a *f*-frequency block as a subset of ≥10 consecutive SNPs. Then, for each such block, we calculate the frequency differential, defined as the absolute difference in mean frequency between the study population and the closer of the LWK and CEU lowlander controls. We focus on regions with large block differential. Specifically, we consider only regions with block differential exceeding the 95% confidence interval (CI) of the sampling variance when sampling *n* = 12 (for Oromos) or *n* = 14 (for Amhara) haplotypes from a population (roughly 20%; see Discussion; Additional file 8). Fifty-eight regions were prioritized after this step.

#### Frequency block differential relative to HapMap control populations

To ensure that the prioritized regions represent positive selection only in Amhara or Oromos highlanders, we expanded our controls to include additional lowlander populations. Specifically, we prioritized regions with block differential exceeding the 95% CI of the sampling variance (see above) compared to all HapMap populations [25]. This ensures we do not consider regions where the dominant haplotype block exists at similar frequency in lowlander populations. Specifically, this helps to avoid spurious signal of selection due to non-African admixture, but also signal that is common between the highlanders and other lowlander populations. Apart from admixture, such common regions may come about independently (due to similar selective constraints), or may be the remnants of selection in an ancestral population. Twenty-seven regions remained prioritized after this step.

#### Frequency block differential after integrating existing genotype data

We also used variant calls from a previous study by Alkorta-Aranburu *et al*. [14] on the same highlander populations. In this study, the authors performed genotyping on 102 Amhara highlanders, and 63 Oromos highlanders. By incorporating the allele frequencies observed in genotyping these larger cohorts, we were able to refine our sample frequencies and identify any false signals that were caused by sampling. For a given region, we extracted all variants from the (previously identified) *f*-frequency block, which were also covered by Alkorta-Aranburu *et al*. We then refined our highlander (Amhara or Oromos) block frequency by taking a weighted average (by sample size) over the observed frequencies in the corresponding population from both studies. For instance, if the observed mean frequency for a haplotype block was 0.8 in our Oromo sample (*n* = 12), and 0.85 in the Oromo sample from Alkorta-Aranburu *et al*. (*n* = 126), the revised block frequency would be set to 0.846. Due to the increased sample size, the 95% CI of sampling error was reduced substantially. Consequently, we prioritized regions where the revised block frequency differential was greater than 10% for all controls. We note that regions that contained no variants sampled by Alkorta-Aranburu *et al*. were unaffected by this criterion. Nineteen regions remained prioritized after this step.

#### RefSeq genes in region

Finally, we prioritized candidate regions that overlap at least one transcript, as defined by RefSeq (release 45, downloaded 14 January 2011). This collection includes protein coding genes, microRNAs, and non-coding RNAs (39,173 transcripts overall). Other regions may contain important regulatory variations; however, for an initial pass, we focused our efforts on regions for which there are more readily accessible methods to identify and validate causal effects.

### Prioritized regions

At the end of this process, only eight regions remained in our list of prioritized regions (see Table 1 for a summary; see Additional file 9 for Manhattan plots). Of these, two were significant in both the Amhara and the Oromos populations. However, due to a lack of overlapping sites from HapMap [25] or from the Alkorta-Aranburu *et al*. study, we were unable to subject one of these loci (chr14:106.32-106.39 M) to the complete battery of prioritization criteria. As a result, we focused on the remaining shared region.

**Table 1:** Significant genomic regions in the Amhara and/or the Oromos populations

	**Chromosomal region**	**A**	**O**	**Tests**	**Genes located in the region**
1	Chr6:29796452-29896452		✓	S_π_, S_f_	*HLA-G*, *HLA-H*, *HCG2P7*, *HCG4P6*
2	Chr9:33915871-34021871		✓	S_π_, S_f_	*UBE2R2*, *UBAP2*, *SNORD121A/B*
3	Chr11:84676260-84910260	✓		S_π_, S_f_	*DLG2*
4	Chr13:78496785-78606785	✓		S_π_	*EDNRB*
5	Chr14:106322845-106396845	✓	✓	F_st_	*KIAA0125*
6	Chr19:42741726-42973726	✓	✓	PBS	*GSK3A*, *ERF*, CIC*, PAFAH1B3*, *PRR19*, *TMEM145*, *MEGF8*, *CNFN*, LIPE*, *CXCL17*
7	ChrX:44982060-45036060		✓	S_π_, S_f_	*CXorf36*
8	ChrX:130614060-130752060		✓	S_π_, S_f_	*OR13H1*

*Genes experimentally validated as affecting hypoxia tolerance in *Drosophila*.

**Figure 1:**
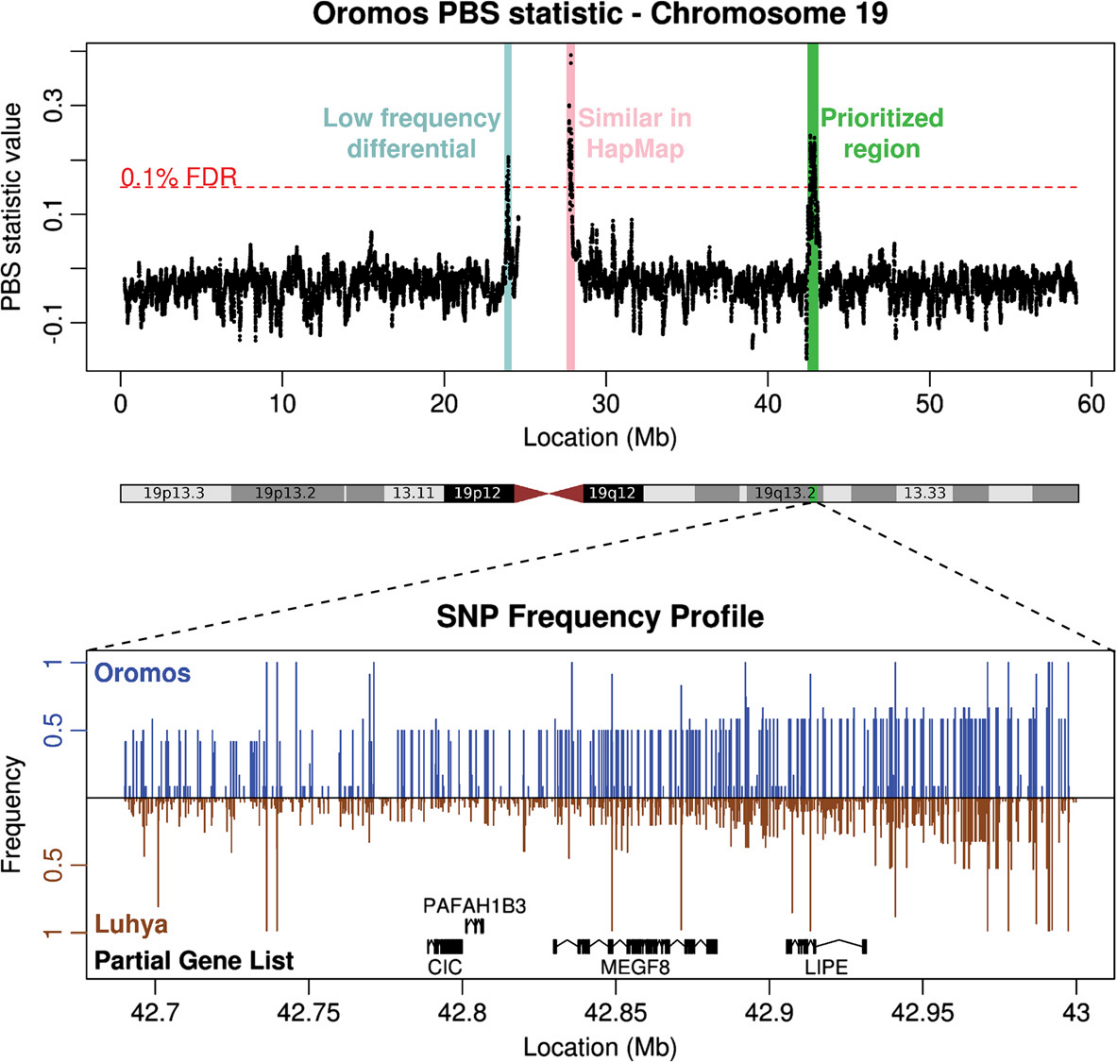
**Population branch statistic (PBS) across chromosome 19 in the Oromos population compared to both the Luhya (LWK) and European (CEU) populations.** The red line represents a genome-wide 0.1% FDR. Three distinct regions exceed this cutoff, two of which are near the centromere and were thus not prioritized. The bottom panel shows the SNP frequency profile in the prioritized region for Oromos (blue) and LWK (brown, inverted). Genes with *Drosophila* orthologs (on which RNAi experiments were conducted) are shown in black below the frequency profiles. As can be seen, variant frequencies in this region are considerably higher in the highlanders than in a nearby lowlander population.

This 208 kbp gene-rich region on chromosome 19 contains a block of 135 'differential' SNPs showing significant change in frequency relative to the control populations (Figure 1). Specifically, the mean variant frequencies are 48% (Oromos), 42% (Amhara), 16% (LWK), and 1% (CEU). We computed the local linkage disequilibrium (LD) structure in the region, and found a strong, and very large, LD block surrounding the region in the Oromos (Additional file 10). A corresponding, but smaller, block was also visible in the Amhara. This is consistent with the longer timespan spent at high altitudes by the Amhara [26], during which recombination may have broken local LD structure.

Of the 10 genes in the region (Table 1), 8 lie in the prioritized region shared across the Oromos and the Amhara. These genes point to many intriguing candidates. For example, the differential SNPs include two missense mutations in the *LIPE* gene (*rs7246232* and *rs16975750*; Additional file 11). While these mutations have not previously been linked to a known phenotype, *LIPE* is associated with gestational hypertension (and consequent placental ischemia) [27]. It belongs to the lipase family, which is known to play a role in hypoxia via lipolysis, triglyceride metabolism, and energy storage [28]. Other genes in this region include *CIC*, which is a transcriptional suppressor involved in early organ development, *CNFN* (involved in hematopoiesis [29]), *CXCL17* (involved in angiogenesis [30]), and *PAFAH1B3* (related to coronary artery disease [31] and organ development [32]). Thus, our results point to a cluster of putative hypoxia response genes. As these genes are associated with phenotypes such as lipid metabolism, transcription regulation, or angiogenesis, they illustrate the potential for a variety of adaptive mechanisms to high altitude in humans.

The remaining seven regions (Table 1) contained several other intriguing gene candidates. For instance, the 110 kbp region on chromosome 13 that is significant for the Amhara population under the S_π_ test contains Endothelin receptor B (*EDNRB*; Additional file 12). This gene encodes a receptor for endothelin, a potent vasoactive peptide, which activates signaling cascades that promote blood vessel constriction [33]. *EDNRB* is also tied into the *HIF* pathway. Specifically, it is a receptor for Endothelin 1, which is directly activated by *HIF*. In addition, it is a known target for drugs (for example, bosentan) prescribed for altitude sickness [34]. In the Amhara population, this gene has 52 fixed, or near-fixed, SNPs (spanning approximately 170 kbp) upstream of the promoter region, 20 of which are in a 10 kbp region containing several transcription-factor binding sites (Additional file 7). As a result, and due to the lack of nonsynonymous coding mutations, we hypothesize that adaptive effects in this region are likely due to regulatory variation. Further study will be needed to determine the mechanism by which this may have occurred. Additionally, we note that the dominant haplotype block is present in the controls, at 36% frequency in LWK and 66% frequency in CEU. Such intermediate frequencies in lowlander controls are consistent with selection acting on standing variation, rather than a *de novo* mutation [35].

### Experimental validation using a model system

To provide further evidence of the role of these genes in hypoxia, we used *D. melanogaster* as a model system to test the hypothesis that differential regulation of their orthologs in flies affects tolerance or susceptibility to low O_2_. Potentially causal variants in the candidate genes may represent either gain- or loss-of-function changes. Due to a lack of nonsynonymous coding variants in most of the genes (with the exception of *LIPE*), we hypothesize that adaptive traits are likely the result of regulatory effects.

**Figure 2:**
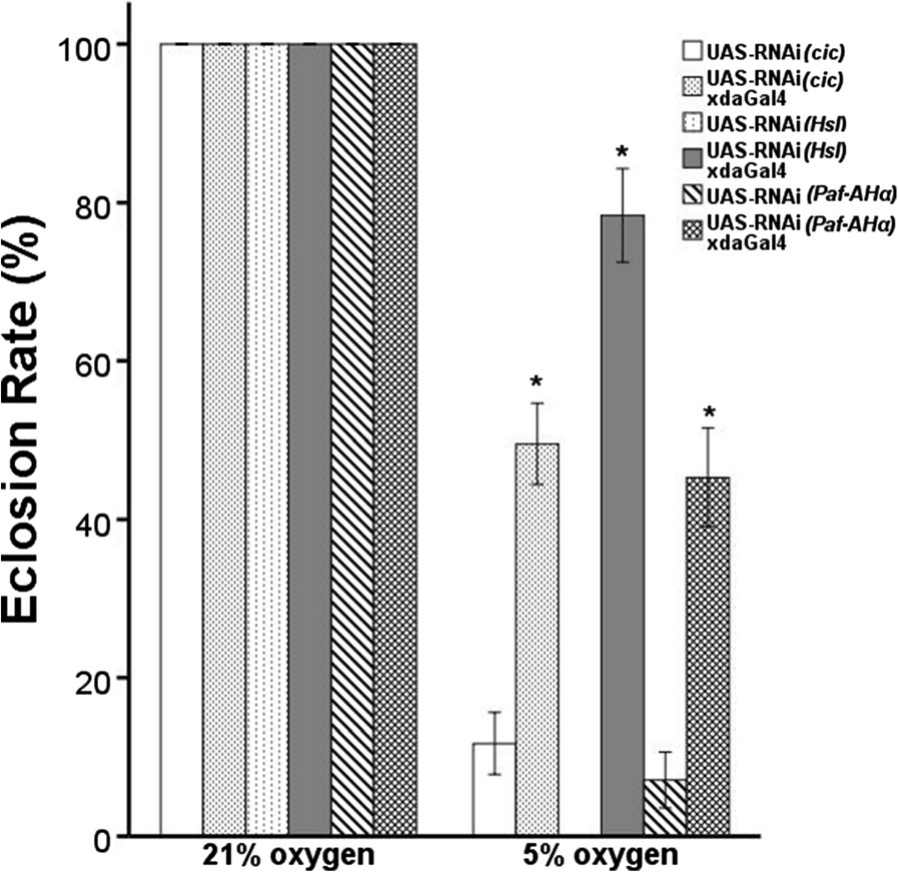
**RNAi-mediated knockdown of candidate human gene orthologs enhanced hypoxia tolerance in *D. melanogaster.*** The available UAS-RNAi lines for *cic* (ortholog of human *CIC*), *Hsl* (ortholog of human *LIPE*) and *Paf-AHα* (ortholog of human *PAFAH1B3*) were crossed with the daughterless (da)-GAL4, a driver strain that expresses GAL4 ubiquitously. The level of hypoxia tolerance was determined by measuring eclosion rate in an atmosphere chamber containing 5% O_2_. The UAS-RNAi stocks without cross were used as a negative control (open bars). Two different UAS-RNAi lines targeting each candidate gene were used in each experiment to minimize off-target effects. Each bar represents the mean ± standard error of the mean value of three separate tests; *P < 0.05.

Because up-regulating a gene may be problematic if it is unexpressed in a particular tissue, we first used the UAS-RNAi/GAL4 system (see Materials and methods) to investigate whether down-regulating the fly orthologs of the candidate genes in the chromosome 19 region has any effect on hypoxia tolerance. Of the eight genes in the region, we tested four (*CIC*, *LIPE*, *PAFAH1B3*, and *MEGF8*) that had *Drosophila* orthologs. Remarkably, three of the four genes, when knocked down, led to markedly improved tolerance to low oxygen. These genes were *cic* (ortholog of human *CIC*), *Hsl* (ortholog of human *LIPE*), and *Paf-AHα* (ortholog of human *PAFAH1B3*). We observed an increase in survival rates that varied from about 40% to 80%, constituting a two- to four-fold increase over controls in the same hypoxic environment (Figure 2).

In contrast, we recently tested 2,187 P-elements fly lines covering 1,870 genes [36] and obtained only 44 lines (approximately 1.5%) showing evidence of tolerance to low O_2_. Assuming this roughly represents the null distribution of random genes affecting hypoxia tolerance, our observation of three of the four tested genes with this effect is statistically significant (*P* = 3.7 × 10^-4^, Fisher’s exact test).

We note that CIC has been shown to function as a repressor of receptor tyrosine kinase (RTK) responsive genes. Following activation of RTK signaling, CIC repression is removed, enabling expression of targeted genes downstream. *CIC* is well conserved from *Drosophila* to humans, and is mostly known in determining cell fate and cell proliferation [37,38]. Of interest is the cross-talk between the RTK and Notch pathways, including core components of the RTK pathway and other major pathways such as transforming growth factor beta, Jak/Stat and Wnt [39]. This is remarkable, as we have previously shown the *Notch* pathway to be crucial for hypoxia tolerance in *Drosophila*[16]. LIPE, a hormone-sensitive lipase, is important in lipolysis and in mobilization of fatty acids and glycerol from fat cells. PAF, a platelet-activating factor, is a potent lipid mediator and is involved in a variety of physiological events. Its deacetylation induces a loss of activity that is catalyzed by PAF-AH, a platelet-activating factor acetyl hydrolase. Type I PAF-AH has two subunits (α and β) and plays a role in cellular functions such as induction of nuclear movement and control of microtubule organization. Further study will be required in order to determine how exactly reduced expression of these transcripts contributes to improved hypoxia tolerance.

Despite other phenotypic differences (see 'Sample description' in Materials and methods), the Amhara and Oromos are both well adapted to high altitudes. Nevertheless, as evident from the chronic mountain sickness scores (Additional file 13), the Amhara appear to be somewhat better adapted. This is consistent with the longer time spent in high altitudes, allowing more opportunity for adaptation to occur. The comparatively shorter time of the Oromos at altitude (600 to 700 years [26]) implies rapid adaptation. One possible explanation for this is selection acting on standing variation, rather than on *de novo* mutations [35]. If true, this may also explain commonalities in adaptation between the populations, as in the chromosome 19 region. An alternative explanation is that a beneficial allele arose in one of the populations, and early migration/admixture carried the haplotype block to the other.

We emphasize that despite this shared region, the majority of the signal for selection is not shared between the two populations (Table 1; Additional file 7). As hypoxia resistance is likely a systematic, complex, and multi-genic trait, we believe that the genes uncovered here explain only part of the adaptive trait, and that further studies in both populations will be required to fully elucidate the adaptive mechanism.

## Discussion

Although the notion that hypoxia tolerance is heritable has dominated high altitude medicine for some time, it was only in recent years that attempts to identify the genetic basis of this adaptation have been made. These studies used genotyping or exome sequencing, but not WGS. The relatively sparse sampling of the genome obtained with these technologies makes it harder to identify shifts in the allele frequency spectrum associated with natural selection. Consequently, many of these studies focused on candidate genes. Moore *et al*. [9] reported one of the first genomic scans for selection in high altitude human populations. Considering roughly 11,000 SNPs, they identified variants in genes related to the hypoxia inducible factor (HIF) pathway, such as Endothelin 1. An extended analysis using genotyping arrays [40] identified candidate genes related to the HIF pathway, including *ETA*, *NOS2A*, and *PRKAA1*. Another study by Simonson *et al.*[11] used LD-based tests, finding signatures of selection in *EGLN1* and *PPARα*. Beall *et al.*[12] identified positive selection in a sample of high-altitude Tibetans at the *EPAS1* locus. Similar studies were also carried out in Ethiopia by Scheinfeldt *et al.*[13], identifying *CBARA1*, *VAV3*, *ARNT2* and *THRB* (the latter two are related to the HIF pathway), by Alkorta-Aranburu *et al.*[14], identifying several hypoxia-related genes (for example, *CUL3*, *ADRBK1*, *CORO1B*), and most recently by Huerta-Sanchez *et al.*[15], identifying a HIF-related gene (*BHLHE41*). Interestingly, we did not observe strong signals of positive selection in our Ethiopian populations for any of these genes. This may be the result of a different assaying technique, as this is the first study in Ethiopians to use WGS in a genome-wide scan for selection. We note that the above-mentioned studies showed no experimental evidence supporting the role of the identified genes in hypoxia tolerance.

There is an important trade-off when comparing WGS to exome sequencing or genotyping studies. Namely, WGS is usually performed on fewer individuals, but provides a near-complete sampling of variant sites. For selection signatures, this is critical. For instance, consider the high frequency block found near the *EDNRB* gene. With WGS, this region corresponds to the highest peak in the chromosome, with a block of 52 variants that are fixed in Amhara, but only 36% in LWK. In contrast, the Nimblegen (Madison, WI, USA) 2.1 M exon capture array targets only two high-frequency variants in this region, none within the block. As for genotyping, the approximately 1 M Affymetrix (Santa Clara, CA, USA) Genome-Wide Human SNP Array 6.0 samples only 2 of the 52 sites in the block, resulting in a much weaker signal (Additional file 14). In both cases, signal in the region did not exceed the respective genome-wide 0.1% FDR, calculated using only the corresponding (exome or genotyping) sites (see Materials and methods). A similar argument holds for seven of the eight final regions (Table 1) identified in our study.

In addition, although genotype imputation is powerful for inferring un-sampled sites, it relies on conserved LD structure between the study population and a reference panel. Yet, positive selection strongly affects the structure of LD in a region by extending haplotype boundaries [41], as is evidently the case in the chromosome 19 region (Additional file 10). As a result, even if LD is generally well conserved between a study population and reference panel, this is less likely to hold in regions affected by positive selection, rendering imputation less effective in genomic scans for selection.

The drawback of sampling fewer individuals is that observed frequency differences may arise from sampling. To account for this, we determined the sampling variance (95% CI) of a SNP at a given frequency that can be expected when sampling 12 to 14 haplotypes from a population (Additional file 8). We then prioritized regions showing a frequency differential that exceeded the sampling variance between highlanders and lowlander controls. Despite this limitation, simulations show that our tests achieve between 67 and 95% power on 12 to 14 haplotypes, compared to a much larger sample of 400 haplotypes (Additional file 15). At the same time, many of the regions we identified would likely have been missed by genotyping studies, implying that WGS represents a complementary approach to sampling-based assays. An optimal study design may, for instance, include WGS of relatively few individuals, followed by targeted sequencing or genotyping of a larger cohort. This would enable a complete genomic scan, as well as increased power derived from larger samples. We achieved a similar design by integrating our WGS data with genotype data from Alkorta-Aranburu *et al.*[14].

## Conclusions

Our study identifies a number of candidate genes for hypoxia tolerance that were not previously reported. To further validate our approach, we tested the impact of down-regulating these genes (using RNAi knock down) on hypoxia tolerance in a *D. melanogaster* model system. Several orthologs, when knocked down, led to increased survival (that is, eclosion) under low O_2_ conditions (two- to four-fold relative to controls). This provides evidence for their important role under hypoxic conditions, and lends further credence to our analysis. Finally, the fact that genes identified by WGS in humans affect flies implies an evolutionarily conserved mechanism for hypoxia tolerance.

## Materials and methods

### Sample description

Ten Oromos subjects from the Bale Plateau in the Oromia region of southeast Ethiopia and seven Amhara subjects from the Chennek field in the Simien Mountains of north Ethiopia were chosen to reflect differences in ancestral adaptation to high altitude. The Oromos generally have darker skin color and a less slender build. They appear more muscular and are generally shorter than the Amhara people. They have lived at high altitudes for 600 to 700 years; a much shorter time compared to the millennia of the Amhara people [26]. The subjects were examined and a history was taken. Only males aged 20 to 40 years found to be free of disease and with a chronic mountain sickness (CMS) score <12 [42] were selected. See Additional file 13 for the complete clinical characteristics of each test subject. Venous blood was obtained in the field, stored, and transported in suitable containers to allow extraction of sufficient DNA from both study populations. Subjects were volunteers, and each subject gave informed written consent in their local language, adhering to a protocol approved by the UCSD institutional review board (IRB00000354).

### DNA extraction, library construction and sequencing

Genomic DNA was isolated using Blood DNA extraction kit (QIAGEN, Valencia, CA, USA) and randomly fragmented. Fragments of the desired length were gel-purified. Adapter ligation and DNA cluster preparation were performed using the library preparation kit according to manufacturer’s instruction (Illumina, San Diego, CA, USA). Whole genome sequencing was performed using Illumina’s Hiseq 2000 platform on all individuals to a mean, per-sample depth of approximately 18× (Additional file 16).

### Read alignment, score recalibration and variant calling

We aligned the reads to the human reference genome (hg19) using BWA [17] with default parameter settings. We adjusted the alignments using GATK indel realignment, Picard read duplicate marking, and GATK quality score recalibration modules [18,19] under default parameter settings, as defined by the GATK manual (version 2). We finally called and filtered the SNPs using the GATK UnifiedGenotyper tool under default settings. As can be seen in Additional file 16, the sequencing was free of any mapping bias in coverage or mapping percentage. As an independent test, we also identified variants using the SoapSNP pipeline [43]. The SoapSNP variants were generally a super-set of the GATK variants, with 25% more calls (9,508,898 versus 7,594,936 for Amhara, and 10,284,853 versus 8,144,023 for Oromos). This is mainly attributed to less restrictive filtering.

### Variant filters

The coverage difference between our study populations (approximately 20×) and lowlander controls (approximately 4×) led to differences in processing the called variants. To adjust for these differences, we filtered our call set using three steps. First, we observed several variants in clustered genomic loci that were discarded by the variant caller in the study (higher coverage) populations. This happens due to various sequencing and mapping artifacts, such as strand bias, low sequence complexity, or structural variations. Due to the low coverage, variants in these loci are not always discarded in the controls. We thus removed from consideration any region comprising 10 consecutive SNPs that were filtered out using GATK in our study population. Second, following the protocol used by the 1000 Genomes Project, we filtered out any site with a mean coverage greater than twice the genome-wide median as likely caused by duplication [21]. This removes variants found in repetitive regions, such as centromeric sequence. We also filtered out any site with less than 2× coverage per person in the study population as being too poorly covered to accurately call SNPs. Finally, we removed sites that had an excess of heterozygotes compared to expectations from Hardy-Weinberg equilibrium. We tested this using a test from Emigh [44] describing the heterozygote probability as:

$$P_{\mathrm{Aa}}=\frac{n!}{n_{\mathrm{AA}}!n_{\mathrm{Aa}}!n_{\mathrm{aa}}!}*\frac{n_{A}!n_{a}!}{\left(2n\right)!}*2^{n_{\mathrm{Aa}}}$$

Variants with *P*-value <0.05 were discarded. After filtering variants based on the three filters described above, we remained with 7,555,907 SNPs in the Amhara population and 8,069,425 SNPs in the Oromos population. See Additional file 17 for an exemplar of the number of variants removed in each filtering step. In addition, to exclude cryptic relatedness in the Oromos and Amhara population samples, we applied PLINK’s [45]$\hat{\pi }$ test for identity by descent. This resulted in excluding four individuals from our initial Oromos sample, finally arriving at six Oromos and seven Amhara individuals.

### Lowlander control populations

To identify appropriate controls, we used low coverage whole-genome sequencing calls from the 1000 Genomes Project populations [21]. We ran ADMIXTURE [20] on 13,928 sampled sites to identify the population most closely related to the highlanders. As seen in Additional file 2, the Ethiopian individuals consist largely of African ancestry, but possess a more substantial European component compared to the other African populations. The closest population consists of 67 Luhya (LWK) individuals from Webuye, Kenya, and was thus chosen as the control for all cross-population tests of selection (see below). As an out-group for the PBS test (see below), we used 90 European (CEU) individuals in order to capture variation in the highlanders shared with individuals of European ancestry. We also performed principle component analysis on our study populations jointly with the lowlander controls (LWK) and out-group (CEU), further illustrating our study populations as an admixture of these two ancestral groups (Additional file 3).

### Identifying regions under positive selection

Under positive selection, haplotypes carrying the beneficial mutation (as well as linked, neutral mutations) rapidly increase in frequency, leading to a loss of genetic diversity in the region surrounding the mutation [46] (illustration in Additional file 5). This loss of diversity, or selective sweep, decreases with distance from the beneficial mutation due to recombination. The loss of allelic diversity and the corresponding skew in the allele frequency spectrum can be used to detect loci important for adaptation to the selective stress [46]. We use cross-population tests to adjust for interesting frequency profiles that are shared between our study and control populations. These are likely due to events (such as bottlenecks, genetic drift, or even selection for a different phenotype) occurring before our study and control populations diverged, and thus likely not related to hypoxia tolerance. Population-specific selection can be measured by comparing the estimated scaled mutation rate θ = 4*N*_e_μ in a given loci to that of the same loci in a control population. A large decrease in θ of the study population compared to controls indicates a region is evolving non-neutrally in the study population, consistent with positive selection. It is important to note that tests of selection may be confounded by several factors, including demographic events (for example, severe bottlenecks) and genetic drift [23]. We therefore use experimental validation in a model system as independent validation that our tests identified adaptive regions.

### Tests of selection

First, we ran two cross-population tests comparing the Amhara or Oromos populations (study) against the 1000 Genomes Luhya population (control). These tests are based on two common estimators of θ: the summed non-fixed frequencies estimator, denoted θ_*f*_, and the average pairwise heterozygosity estimator, denoted θ_π_[47]. For a given region, a high log ratio of θ_π_ (θ_f_) in the control relative to the study population is indicative of selection [48]. We label these log ratio statistics as S_π_ for the average heterozygosity estimator and S_f_ for the summed frequency estimator, such that:

$$S_{f}=\log\;\left(\frac{\theta_{f,\mathrm{control}}}{\theta_{f,\mathrm{study}}}\right)$$

$$S_{\pi }=\log\;\left(\frac{\theta_{\pi ,\mathrm{control}}}{\theta_{\pi ,\mathrm{study}}}\right)$$

Another class of tests for selection is based on the fixation index, or F_st_, between two populations [49]. This class aggregates differential SNP frequencies across two populations. For instance, Hudson *et al.*[50] define this measure as:

$$F_{\mathrm{st}}=1-\frac{\pi_{w}}{\pi_{b}}$$

Where π_w_ represents the within-population average heterozygosity and π_b_ represents the between-population average heterozygosity. As two populations diverge, the variability between the populations increases much more than the variability within each population, and the statistic approaches one. The fixation index roughly correlates to the evolutionary branch length *T* between two populations [51] as:

$$T=-\log\left(1-F_{\mathrm{st}}\right)$$

This approach is not directional, however. As a result, a significant statistic value may indicate a selective sweep in either the study or the control population. To address this, Shriver *et al.*[52] and Yi *et al.*[10] developed the concept of a population branch statistic, or PBS. This combines the pairwise branch lengths of three populations as follows:

$$\mathrm{PBS}=\frac{T^{\mathrm{SN}}+T^{\mathrm{SO}}-T^{\mathrm{NO}}}{2}$$

Where S represents a study population, N represents an evolutionarily close control population, and O represents a distant out-group. We calculated the PBS with our study population defined as either the Amhara or Oromos population, our control as the Luhya population, and our out-group as the CEU population. Additionally, we compared the results of the above tests with XP-CLR [53], a method that attempts to detect large linkage blocks with high frequency differential as indicative of positive selection.

For S_f_, S_π_, F_st_, and PBS, we use genomic windows of size 50 kbp, overlapping at 2 kbp intervals. For each test, we define the top 0.1% genome-wide value as the genomic-control cutoff to determine the windows of interest. The code used to compute these test statistics can be downloaded from [54].

For the XP-CLR test statistic, we found that using a 0.1% genome-wide threshold was overly stringent. Testing Amhara versus Luhya using a 0.1% threshold, exactly five non-overlapping regions exceeded the threshold, all of which contained highly repetitive sequence (except for the HLA region, which has a high mutation rate). Relaxing the threshold to 0.3% genome-wide yielded a comparable number of regions to that found by our other tests, but since XP-CLR uses variable size genomic windows (normally much larger than 50 kbp), the list of implicated genes was dominated by XP-CLR results. Hence, we used XP-CLR only for secondary validation. For instance, the EDNRB gene region on chromosome 13 was found to be significant using XP-CLR under a 0.3% threshold.

### Population simulations and power estimation

We generated simulated populations using the *mpop* forward simulator [55] and Hudson’s *ms* coalescent simulator [56]. For a given set of parameters μ, *r*, *s*, *t* (mutation rate, recombination rate, selection coefficient and time since selection, respectively) we generated 200 sets of simulated populations. We initiated each instance with a unique source population of N_e_ = 1,000 diploids from a neutral coalescent process, using Hudson’s *ms* simulator. We then sampled with replacement from the source population into three separate populations of size *N*_*e*_ each, labeled 'study', 'cont1', and 'cont2'. We evolved these populations separately using the *mpop* simulator, such that only the study population had a locus under positive selection. Individuals carrying the advantageous allele had higher likelihood (∝ 1 + *s*, for a homozygous carrier) to reproduce at each generation. The other populations (*cont1* and *cont2*) continued to evolve neutrally. After τ generations, a random sample (*n* = 100 diploids) was taken from each of the three populations, and cross-population neutrality tests were applied. Genomic regions of size 50 kbp were simulated, with mutation and recombination rates set to *μ* = 2.4 × 10^-7^ and *r* = 3.784 × 10^-8^ per base, per generation. The selection coefficient used for these simulations was *s* = 0.02, and the number of generations since selection τ ranged between [50, 4000].

The power of a test statistic at 5% false positive rate was determined as the fraction of statistic values exceeding a certain cutoff when applied to the study versus cont1 samples. Cutoff values were set to the top 5% of the null distribution, obtained by applying the same test to samples from two simulated populations evolving neutrally (cont1 versus cont2).

### Power of different tests under varying model parameters

The different tests for selection described above all aim to find regions with marked differences in allele frequencies across study and control populations. However, the specific signal observed is highly influenced by different factors, such as the selection coefficient and the time since selection. In general, the allelic divergence in a region is a function of the local mutation and recombination rates. Under a Wright-Fisher model of neutral evolution, the expected distribution of allele frequencies (the site frequency spectrum) is known. Specifically, the expected number of alleles with frequency *f*, where *f* = (1,…,*n*-1), is given by *θ*/*f*[47]. Under selective pressure, the site frequency spectrum begins to shift [57]. Initially, the haplotypes carrying the beneficial alleles rapidly increase in frequency, reducing the overall divergence (a selective sweep). Shortly after the beneficial allele becomes fixed in the population, the divergence is at its lowest, and the signal of selection is strongest. As time passes, *de novo* mutations and recombination events gradually restore variability to the region. Initially, there is an increase in low frequency alleles, which then reach intermediate and high frequencies, finally drowning out the selection signal. Thus, there are three major regimes for a population under positive selection: 'pre-fixation', where the beneficial haplotype starts to rise in frequency; 'near fixation', where the haplotype approaches fixation; and 'post-fixation', where the haplotype is fixed in the population, and *de novo* mutations slowly restore diversity to the population. In these regimes, the four tests show different relative strengths in detecting positive selection (Additional file 4). Importantly, although in the example shown the selection coefficient was set to *s* = 0.02, the performance of the different tests diverges further under different selection pressures (where some tests dominate in weaker selection and others in stronger selection) as well as under different demographic histories [22].

#### S_f_ test

The S_f_ test sums non-fixed frequencies in a region, effectively down-weighting low frequency alleles [58]. The result of this is that the S_f_ test is optimized for the post-fixation regime, since in this regime, the bulk of the signal comes from the reduced number of intermediate and high frequency alleles in the study population. As *de novo* mutations only approach these frequencies many generations after fixation, S_f_ is able to detect selection for longer periods of time after fixation has occurred than other tests. In addition, S_f_ excels at capturing stronger selection pressures, where the beneficial allele goes to fixation relatively quickly and thus reaches the post-fixation regime sooner. As Additional file 4 shows, once in the post-fixation regime, the peak power for S_f_ is sustained for more generations compared to other tests.

#### S_π_ test

The S_π_ test is similar to S_f_ in that it is sensitive to strong selection pressures, as well as long times since selection start. However, S_π_ is based on average heterozygosity, which weights allelic differences identically (in other words, S_π_ returns the same value if the derived allele is defined as either the major or the minor allele). This essentially folds the frequency spectrum [58], leading to two major benefits. First, an approach such as S_f_ depends heavily on the idea that the ancestral allele is the reference nucleotide. If this is not true, for instance, a variant at 10% frequency can be mistaken for a variant at 90% frequency instead, heavily impacting the statistic value. For a folded spectrum, however, this is not the case. In addition, unlike S_f_, S_π_ can detect the loss of diversity due to a loss of intermediate frequency alleles, causing it to pick up selection prior to fixation (where there is an abundance of high frequency alleles). However, since it folds the spectrum, S_π_ cannot distinguish high frequency from low frequency variants, and thus only has high power until *de novo* mutations reach intermediate frequencies.

#### F_st_ and PBS tests

As mentioned previously, under positive selection, as the beneficial haplotype dominates the study population, the variability within this population decreases. This can be tested directly using the relative allele frequency spectra as in S_f_ and S_π_, but there is additional information in the site-specific frequency differences across the study and control populations. For instance, let us consider a variant at 20% frequency in the control population. In the study population, this variant lies on a beneficial haplotype, and is sampled at frequency 80%. Under S_π_, this variant contributes equally to both the study and the control statistics, while in S_f_, this variant contributes negatively to the overall statistic. However, there is clearly a sharp rise in frequency, representing an increased branch length between the study and control populations in the phylogenetic tree, which may be indicative of selection. Since the F_st_ test measures the site-specific variability between populations (π_b_), it would be able to detect such situations. Importantly, the scenario described above is consistent with selection occurring on standing variation, where the beneficial haplotype is present in non-negligible frequencies in the control population. However, the undirected nature of the branch lengths presents disadvantages. For instance, a significant F_st_ value could also indicate positive selection in the control population. This is addressed in the PBS test by calculating population-specific branch lengths using multiple controls.

### Effects of sample size on power

In our study, we performed high coverage (15 to 20×), WGS on seven Amhara and six Oromos individuals. Alternative approaches to WGS would include exome sequencing or genotyping. As these approaches are currently less expensive, this may allow for sampling more individuals. In Additional file 15, we show the impact of sample size on power, using simulated populations. The simulation procedure was similar to the one described above, with 500 neutral initial populations, and selection coefficient fixed at *s* = 0.02. As previously described (Additional file 5), there appear to be three general regimes of selection ('pre-fixation', 'near fixation', and 'post-fixation'), where different tests vary in their relative performance across regimes. We focus on a single test, S_π_, and sample from each of the three regimes (τ = 450, 700, 1,000, 1,500 generations after selection begins). In order to identify the effect of decreasing the sample size on power, we vary the sample sizes from *n* = (2, …, 40). As a gold standard for maximal attainable power, we used a large sample size of *n* = 400. Although sequencing more individuals would improve the sensitivity, as seen in Additional file 15, sampling 12 or 14 haplotypes yields between 67 and 95% power compared to our gold standard. Notably, we see that sampling fewer individuals has the greatest impact in the 'pre-fixation' regime. This is due to two factors. First, sampling fewer individuals leads to higher variance in the observed frequencies (Additional file 8). Second, the pre-fixation regime is when the frequency differential of the beneficial haplotype block compared to controls is lowest. Despite this, we are still able to detect positive selection in the majority of our simulated cases.

### Comparison of whole genome sequencing with other assays

We also tested the power of whole genome sequencing in comparison to other technologies, due to its unique ability to capture all allelic variation in a region. Additional file 14 shows the S_π_ test applied to chromosome 13, which contains one of our top hits (the *EDNRB* gene region). We compared the variants captured in our study to those captured by two alternative approaches: whole exome sequencing and genotyping. To mimic the effects of whole exome sequencing, we masked variants not targeted by the Nimblegen 2.1 M exon capture array. For comparison with genotyping studies, we masked variants not included in the approximately 1 M Affymetrix Genome-Wide Human SNP Array 6.0. As shown in Additional file 14, with WGS the strongest signal chromosome-wide is located in the *EDNRB* gene region. In contrast, genotyping shows a significantly weaker signal in the region, while whole exome sequencing shows no signal at all. Thus, for situations where a large portion of the signal is in intergenic or intronic sequence, WGS may provide a major advantage over other technologies.

### *Drosophila* stocks and test of hypoxia tolerance

The *D. melanogaster* stocks carrying UAS-RNAi transgene were obtained from the Vienna *Drosophila* RNAi Center (Vienna, Austria; stock numbers 25995 and 103805 carrying UAS-RNAi(*cic*); 22358 and 109336 carrying UAS-RNAi(*Hsl*); 29003 and 107333 carrying UAS-RNAi(*Paf-Ahα*); 42462 and 8018 carrying UAS-RNAi(*CG7466*)). The da-Gal4 driver (stock number 8641) was obtained from Bloomington stock center (Bloomington, IN, USA).

Hypoxia tolerance of *Drosophila* crosses with specific RNAi-mediated knockdown was carried out as described in [16]. Fifteen virgin female flies homozygous for UAS-RNAi were crossed with 10 male flies homozygous for da-GAL4 and allowed to lay eggs for 24 hours in normoxia. The vials with the eggs were transferred into a computer-controlled atmosphere chamber supplied with 5% oxygen balanced with nitrogen, with 12 hour-dark and 12 hour-light cycle at 22 ± 1°C. The Gal4 driver and UAS-RNAi stocks alone without crossing were included in parallel as controls. After three weeks of culturing, the vials were assayed for the number of pupal cases that were empty or full to calculate the eclosion rate. Six vials of each condition were completed in 2 different experiments for a minimum of 200 pupal cases scored for each condition/cross. The eclosion rate was presented as percentage of empty pupae in all scored pupal cases.

### Data availability

Genotype data have been deposited in dbGaP under accession number phs000647.v1.p1.

## Abbreviations

bp: base pair; CI: confidence interval; FDR: false discovery rate; HIF: hypoxia inducible factor; LD: linkage disequilibrium; PBS: population branch statistic; RNAi: RNA interference; SNP: single nucleotide polymorphism; WGS: whole genome sequencing.

## Competing interests

The authors declare that they have no competing interests.

## Authors’ contributions

DZ, VB, and GGH designed the experiments with input from NU, RR, JX, and KAF. NU, RR, and VB performed sequence and variation analysis, including tests for positive selection. JL, YY, YD, LG, RC, YW, XJ, CH, WJ, DC, GG, SL, and YL performed whole genome sequencing and read alignment. TS, JX, and DZ performed hypoxia tolerance analysis in *Drosophila*. OA, VEC, RH, JLG, MZ, and GZ collected human blood samples. NU, RR, DZ, VB, and GGH wrote the manuscript with comments from co-authors. NU, RR, DZ, VB, and GGH contributed equally to the project. All authors read and approved the final manuscript.

## Supplementary Material

Additional file 1: Figure S1 Computational analysis workflow. The raw reads were mapped using BWA, followed by indel realignment, duplicate marking, and quality score recalibration using the GATK pipeline. Variants were then called and filtered using GATK’s UnifiedGenotyper. After applying additional variant filters to account for the differences in coverage between the study and control populations, we applied several complementary tests to identify 420 regions as candidates for positive selection. Of these, 412 were filtered using 4 prioritization filters customized to the challenges of our sequencing framework, leading to 8 final prioritized regions. Click here for file

Additional file 2: Figure S2 ADMIXTURE analysis with six clusters on the Ethiopian highlanders, along with the 1000 Genomes populations. The highlander ancestry is a mixture of traditionally African and traditionally European genotypes, represented by the green and dark blue segments, respectively. Within the African 1000 Genomes populations, the nearest population geographically as well as ancestrally appears to be the Luhya (LWK) population. We thus selected this population as our control. Similarly, the section sharing ancestry with European populations appears closer to the southern and western Europeans than the Finnish population. As a result, as outgroup in the PBS test, we used the CEU population. Click here for file

Additional file 3: Figure S3 Principal component analysis of the Ethiopian highlanders, along with the 1000 Genomes control (LWK) and out-group (CEU). As can be seen, the first principal component separates the four population samples, further illustrating that the Amhara and Oromos highlanders posses a mixture of African and European ancestry. Click here for file

Additional file 4: Figure S4 Power of neutrality tests used in this study (S_π_, F_st_, S_f_, and PBS) as function of time. **(A)** The x-axis scales linearly in terms of generations since selection start. **(B)** Power as function of logarithmically scaled time for the neutrality tests used in this study. We also show the x-axis in units of *ln(2Ns)/s* (top axis), which can define the regimes as a function of selection pressure. We observe three major regimes, corresponding to the state of the beneficial haplotype in the case population: before the haplotype has significantly risen in frequency ('pre-fixation'), as the haplotype dominates the case population ('near fixation'), and after the haplotype has gone to fixation, while the frequency spectrum gradually reverts to neutrality ('post-fixation'). In these three regimes, the statistics perform differently: PBS performs better in the first regime, S_π_ performs best in the second regime, and S_f_ dominates the third regime. Click here for file

Additional file 5: Figure S5 Illustration of a selective bottleneck in one of two diverged populations, leading to a loss of genetic diversity. The haplotype carrying the beneficial allele (shown in blue) becomes dominant in the population under selection, at the expense of other haplotypes that die out (black lines near the selective bottleneck). This leads to decreased genetic diversity, characterized by a skew in the site frequency spectrum (top) relative to neutrality (bottom). As time progresses, genetic diversity is gradually restored to the region via *de novo* mutation (seen in the 'post-fixation' regime). Click here for file

Additional file 6: Table S1 Regions identified as significant under a 0.1% genome-wide false discovery rate. Click here for file

Additional file 7 **Supplementary Dataset 1.** Regions exceeding the 1% genome-wide FDR for the four tests of selection (S_π_, S_f_, F_st_, and PBS) and the final list of prioritized regions. In addition, it contains a list of nonsynonymous SNPs within the final regions, as well as SNPs in ENCODE and TRANSFAC transcription factor binding sites overlapping these regions. Provided as a separate Excel file with the tables as sheets. The file can be viewed with Microsoft Excel Viewer. Click here for file

Additional file 8: Figure S6 The impact of sampling haplotypes from a population on observed allele frequencies. The red (blue) line shows the 95% confidence interval (CI) of observed frequency when sampling n = 12 (n = 14) haplotypes from a population. This corresponds to our Oromos and Amhara population samples, respectively. For most intermediate frequencies, a difference of around 20% is within the 95% CI. We use the 95% CI frequency difference as a cutoff, prioritizing regions containing haplotype blocks with a greater frequency differential between the highlander population and lowlander controls. For regions on the X chromosome, the number of sampled haplotypes is half, and we therefore required a greater frequency differential (approximately 40% for intermediate frequencies). Click here for file

Additional file 9 **Test statistic values on chromosomes 1 to 22, as well as X, in the Amhara and the Oromos populations.** The tests shown are PBS, F_st_, S_π_, and S_f_. Regions exceeding the 0.1% genomic FDR and that passed all prioritization criteria are shown in green. Click here for file

Additional file 10: Figure S7 Linkage disequilibrium (LD) near the chromosome 19 region. In Oromos (top left), Amhara (top right), as well as the two 1000 Genomes lowlander controls: LWK (bottom left) and CEU (bottom right). The center of the region is marked by a black star. We observe a strong, and large, LD block surrounding the chromosome 19 region in Oromos. A corresponding, but smaller, block is also visible in the Amhara. This observation is in line with the longer time spent at high altitude by the Amhara population, during which recombination may have broken local LD structure. We note that the overall higher levels of LD observed in the Oromos and Amhara may be due to smaller sample sizes in these populations, but that this should be mostly a background effect, and is thus not expected to significantly alter the observed block structure. Click here for file

Additional file 11: Table S2 Nonsynonymous SNPs with significant frequency differential in one of the eight prioritized regions. Click here for file

Additional file 12: Figure S8 Evidence supporting *EDNRB* as a gene candidate. Top panel: S_π_ statistic values across chromosome 13 in the Amhara population, compared to the Luhya (LWK) population. The red line represents a genome-wide, 0.1% FDR. Two distinct regions exceed this cutoff, one of which did not show a haplotype block with significant frequency differential compared to our lowlander controls, and was thus removed from consideration. Bottom panel: SNP frequency profile of the significant region in the Amhara (blue) compared to Luhya (brown, inverted) populations. As can be seen, variant frequencies in this region are much higher in the Amhara population compared to lowlander controls. Click here for file

Additional file 13: Table S3 Clinical characteristics of Oromos and Amhara subjects. Click here for file

Additional file 14: Figure S9 Impact of whole genome sequencing on selection signals. **(A-C)** S_π_ statistic values across chromosome 13 in the Amhara population compared to the Luhya (LWK) population, using the complete set of variants from whole genome sequencing (A), the subset that overlap targets from whole exome capture (B), and the subset (about 1 M) that overlaps a popular genotyping array (C). The red lines represent the respective genome-wide 0.1% FDR calculated individually for each case. Highlighted in green is the *EDNRB* gene loci. **(D-F)** SNP frequency profiles of the *EDNRB* region in Amhara (blue) compared to Luhya (brown, inverted) for whole genome sequencing (D), whole exome sequencing (E), and genotyping (F). As can be seen from the green highlighted regions (A-C), the strong signal present when considering whole genome sequencing is reduced drastically with genotyping and is entirely absent with exome sequencing. Click here for file

Additional file 15: Figure S10 The impact of sequenced sample size on power, using S_π_ as an exemplar test. Five hundred populations were simulated with a fixed selection coefficient of *s* = 0.02 and sampled at different times after selection start. Sample size is shown in haplotypes, and ranges in *n* = (2,3,…,40). Optimal power at each time was determined using a large sample size of *n* = 400. The populations were sampled at four time points representing each of the observed regimes: *t* = 450 for 'pre-fixation', *t* = 700 and *t* = 1,000 for 'near-fixation', and *t* = 1,500 for 'post-fixation'. Although we see an increase in power as more haploptypes are sampled, sampling 12 or 14 haplotypes (our Oromos and Amhara populations, respectively) yields 67 to 95% of the optimal power. Click here for file

Additional file 16: Table S4 Sequencing depth and coverage statistics per individual in the sample. Click here for file

Additional file 17: Table S5 Number of variants removed in each filtering step, for the Luhya and Oromos populations. Click here for file
