# Supplementary figures and images for: Whole genome sequencing of Ethiopian highlanders reveals conserved hypoxia tolerance genes
Source: Genome Biol. 2014 Feb 20;15(2):R36. doi: 10.1186/gb-2014-15-2-r36 (PMC4054780; doi:10.1186/gb-2014-15-2-r36)

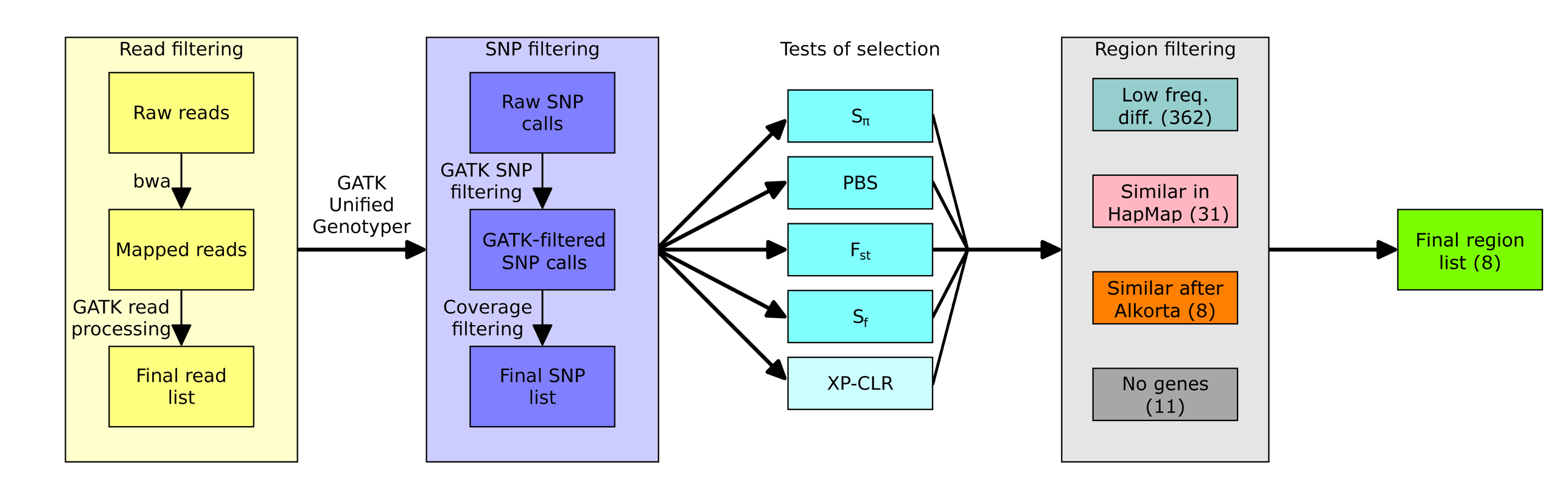

Supplement: Additional file 1: Figure S1 — Computational analysis workflow. The raw reads were mapped using BWA, followed by indel realignment, duplicate marking, and quality score recalibration using the GATK pipeline. Variants were then called and filtered using GATK’s UnifiedGenotyper. After applying additional variant filters to account for the differences in coverage between the study and control populations, we applied several complementary tests to identify 420 regions as candidates for positive selection. Of these, 412 were filtered using 4 prioritization filters customized to the challenges of our sequencing framework, leading to 8 final prioritized regions. [file gb-2014-15-2-r36-S1.png]

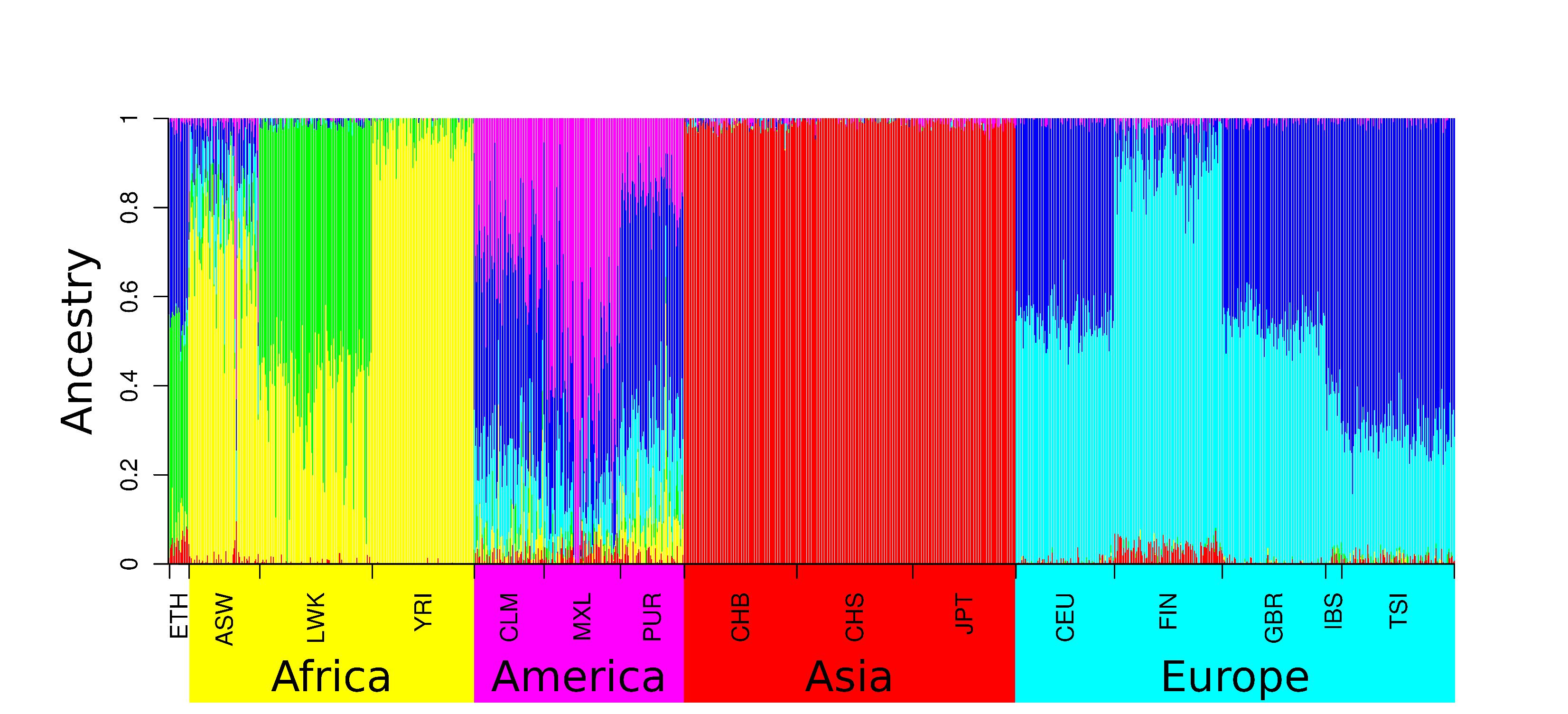

Supplement: Additional file 2: Figure S2 — ADMIXTURE analysis with six clusters on the Ethiopian highlanders, along with the 1000 Genomes populations. The highlander ancestry is a mixture of traditionally African and traditionally European genotypes, represented by the green and dark blue segments, respectively. Within the African 1000 Genomes populations, the nearest population geographically as well as ancestrally appears to be the Luhya (LWK) population. We thus selected this population as our control. Similarly, the section sharing ancestry with European populations appears closer to the southern and western Europeans than the Finnish population. As a result, as outgroup in the PBS test, we used the CEU population. [file gb-2014-15-2-r36-S2.png]

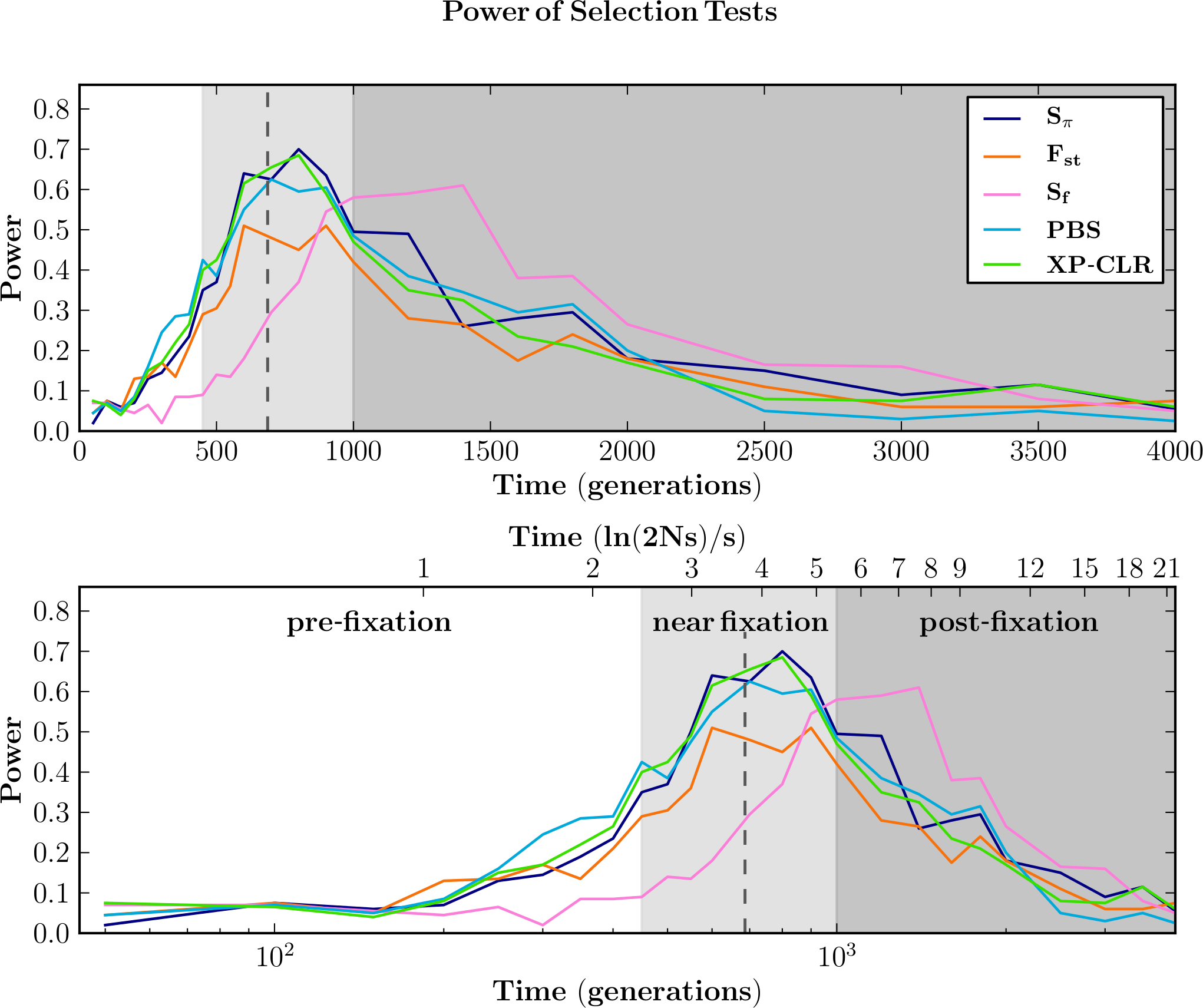

Supplement: Additional file 4: Figure S4 — Power of neutrality tests used in this study (Sπ, Fst, Sf, and PBS) as function of time. (A) The x-axis scales linearly in terms of generations since selection start. (B) Power as function of logarithmically scaled time for the neutrality tests used in this study. We also show the x-axis in units of ln(2Ns)/s (top axis), which can define the regimes as a function of selection pressure. We observe three major regimes, corresponding to the state of the beneficial haplotype in the case population: before the haplotype has significantly risen in frequency ('pre-fixation'), as the haplotype dominates the case population ('near fixation'), and after the haplotype has gone to fixation, while the frequency spectrum gradually reverts to neutrality ('post-fixation'). In these three regimes, the statistics perform differently: PBS performs better in the first regime, Sπ performs best in the second regime, and Sf dominates the third regime. [file gb-2014-15-2-r36-S4.png]

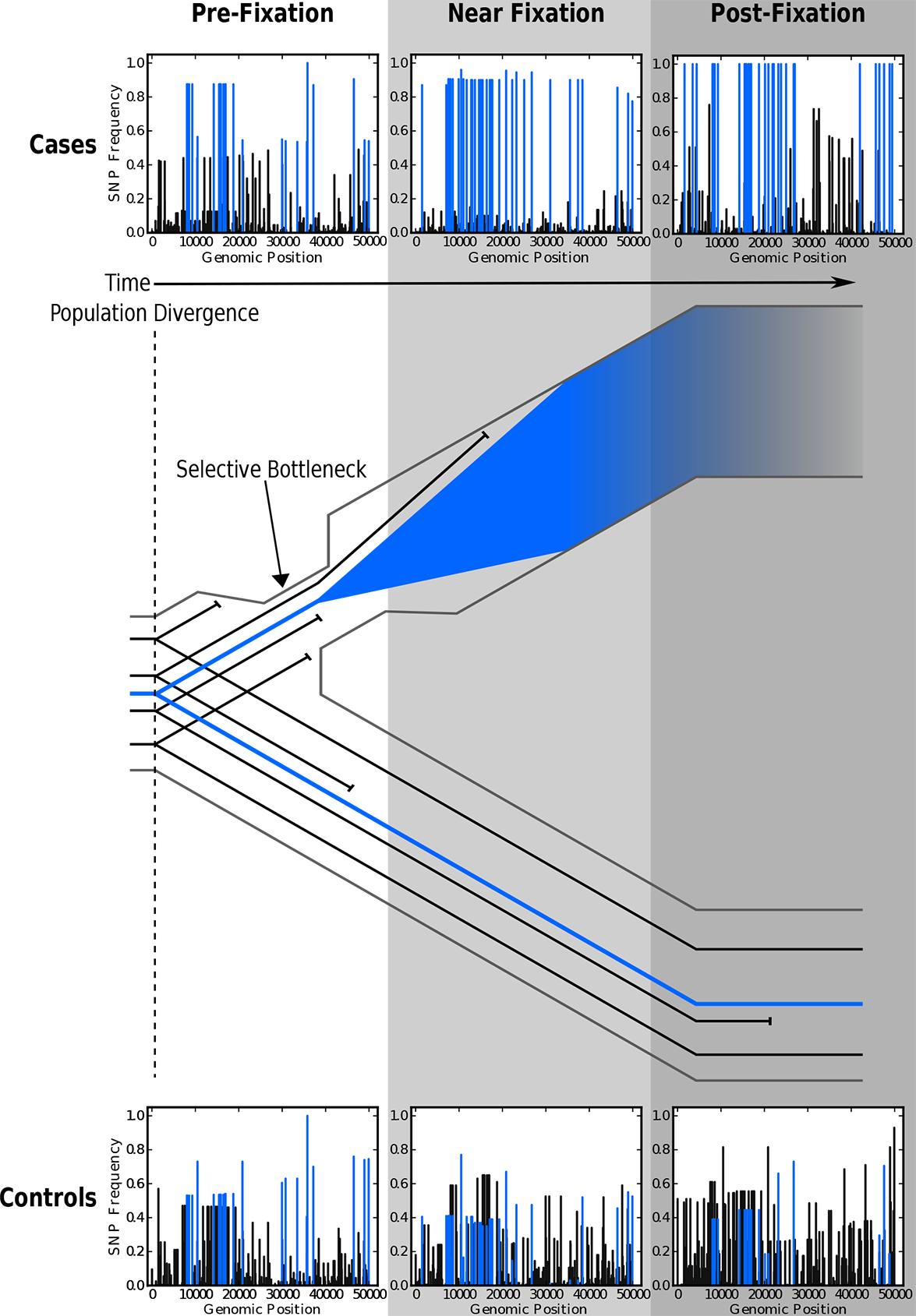

Supplement: Additional file 5: Figure S5 — Illustration of a selective bottleneck in one of two diverged populations, leading to a loss of genetic diversity. The haplotype carrying the beneficial allele (shown in blue) becomes dominant in the population under selection, at the expense of other haplotypes that die out (black lines near the selective bottleneck). This leads to decreased genetic diversity, characterized by a skew in the site frequency spectrum (top) relative to neutrality (bottom). As time progresses, genetic diversity is gradually restored to the region via de novo mutation (seen in the 'post-fixation' regime). [file gb-2014-15-2-r36-S5.png]

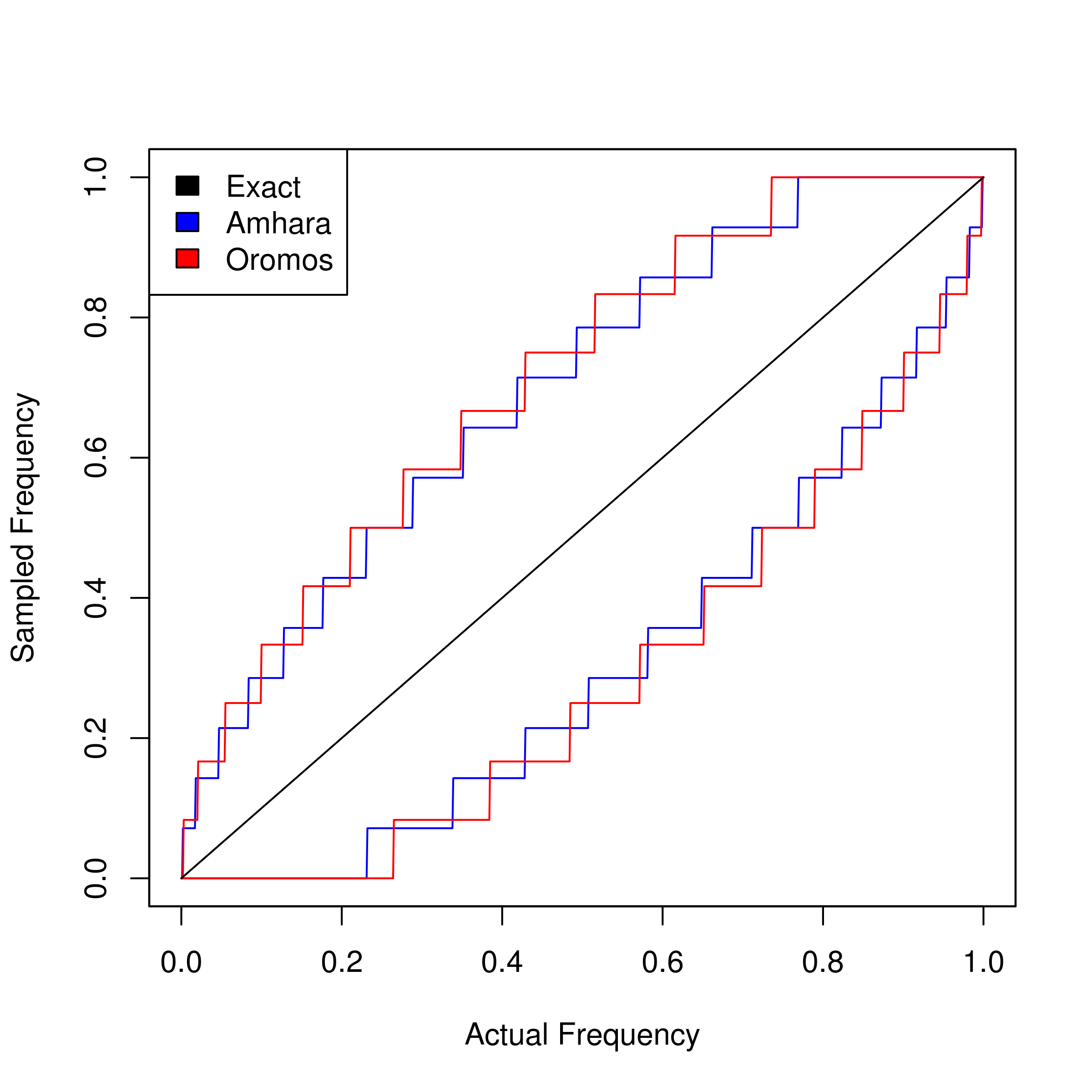

Supplement: Additional file 8: Figure S6 — The impact of sampling haplotypes from a population on observed allele frequencies. The red (blue) line shows the 95% confidence interval (CI) of observed frequency when sampling n = 12 (n = 14) haplotypes from a population. This corresponds to our Oromos and Amhara population samples, respectively. For most intermediate frequencies, a difference of around 20% is within the 95% CI. We use the 95% CI frequency difference as a cutoff, prioritizing regions containing haplotype blocks with a greater frequency differential between the highlander population and lowlander controls. For regions on the X chromosome, the number of sampled haplotypes is half, and we therefore required a greater frequency differential (approximately 40% for intermediate frequencies). [file gb-2014-15-2-r36-S8.png]

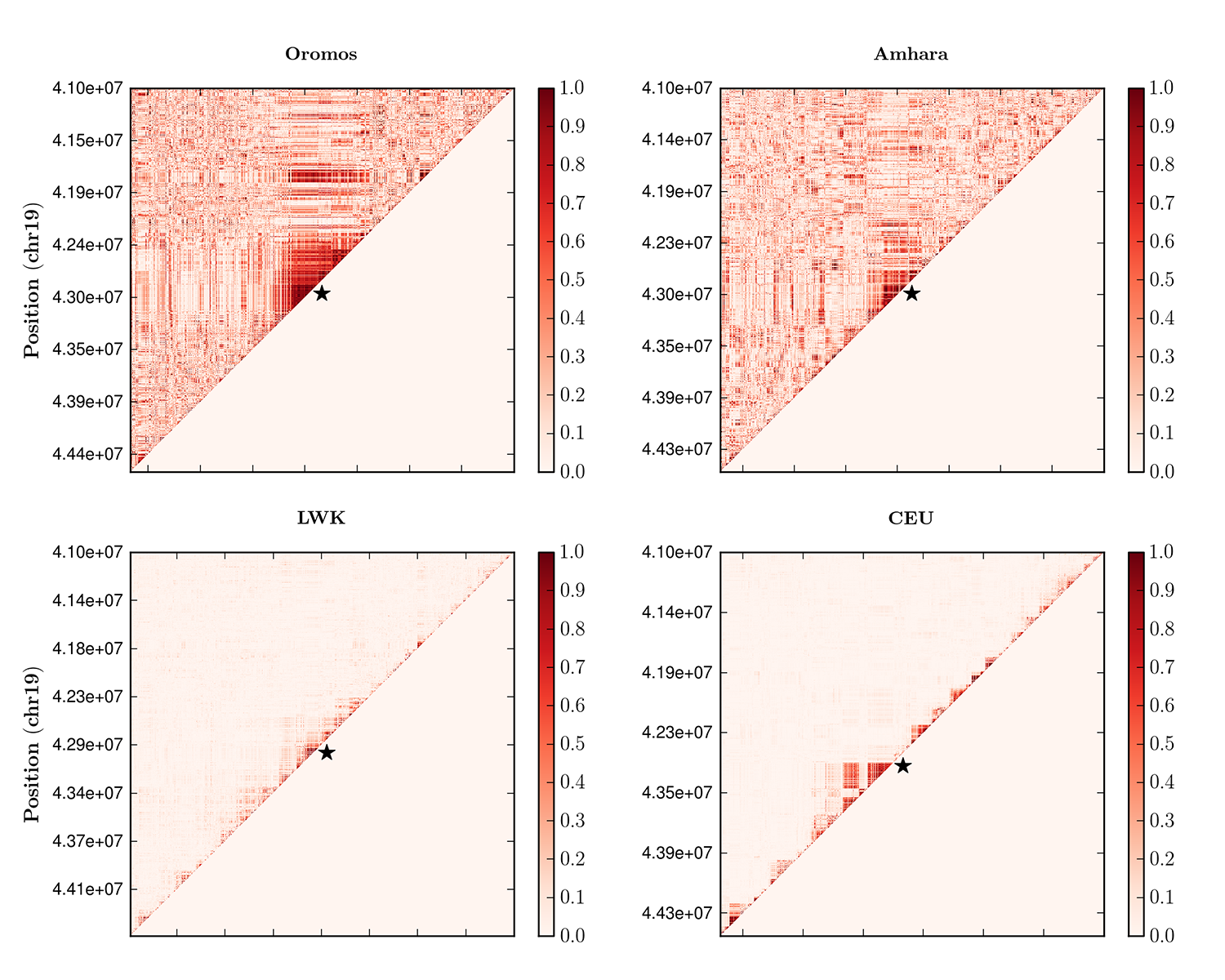

Supplement: Additional file 10: Figure S7 — Linkage disequilibrium (LD) near the chromosome 19 region. In Oromos (top left), Amhara (top right), as well as the two 1000 Genomes lowlander controls: LWK (bottom left) and CEU (bottom right). The center of the region is marked by a black star. We observe a strong, and large, LD block surrounding the chromosome 19 region in Oromos. A corresponding, but smaller, block is also visible in the Amhara. This observation is in line with the longer time spent at high altitude by the Amhara population, during which recombination may have broken local LD structure. We note that the overall higher levels of LD observed in the Oromos and Amhara may be due to smaller sample sizes in these populations, but that this should be mostly a background effect, and is thus not expected to significantly alter the observed block structure. [file gb-2014-15-2-r36-S10.png]

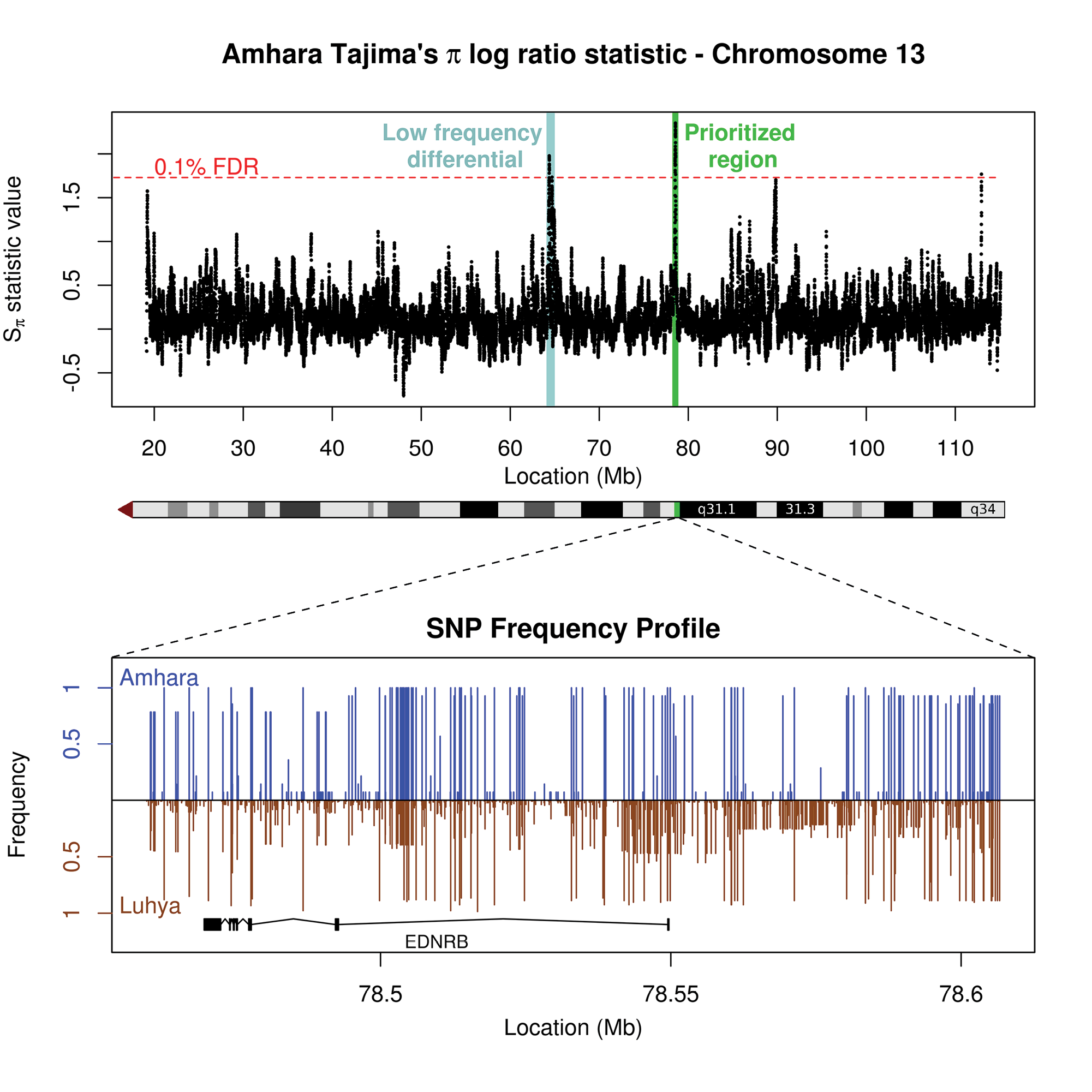

Supplement: Additional file 12: Figure S8 — Evidence supporting EDNRB as a gene candidate. Top panel: Sπ statistic values across chromosome 13 in the Amhara population, compared to the Luhya (LWK) population. The red line represents a genome-wide, 0.1% FDR. Two distinct regions exceed this cutoff, one of which did not show a haplotype block with significant frequency differential compared to our lowlander controls, and was thus removed from consideration. Bottom panel: SNP frequency profile of the significant region in the Amhara (blue) compared to Luhya (brown, inverted) populations. As can be seen, variant frequencies in this region are much higher in the Amhara population compared to lowlander controls. [file gb-2014-15-2-r36-S12.png]

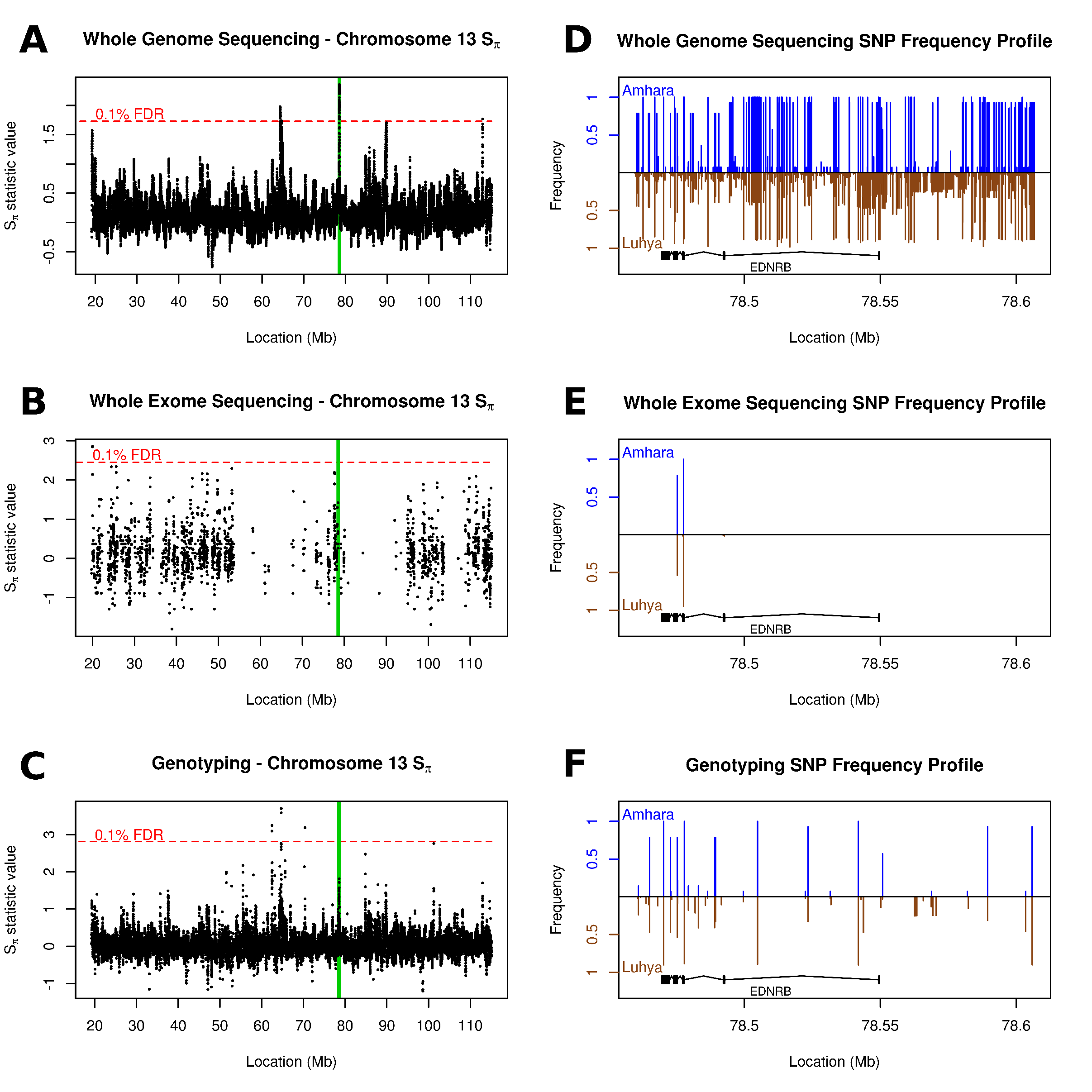

Supplement: Additional file 14: Figure S9 — Impact of whole genome sequencing on selection signals. (A-C) Sπ statistic values across chromosome 13 in the Amhara population compared to the Luhya (LWK) population, using the complete set of variants from whole genome sequencing (A), the subset that overlap targets from whole exome capture (B), and the subset (about 1 M) that overlaps a popular genotyping array (C). The red lines represent the respective genome-wide 0.1% FDR calculated individually for each case. Highlighted in green is the EDNRB gene loci. (D-F) SNP frequency profiles of the EDNRB region in Amhara (blue) compared to Luhya (brown, inverted) for whole genome sequencing (D), whole exome sequencing (E), and genotyping (F). As can be seen from the green highlighted regions (A-C), the strong signal present when considering whole genome sequencing is reduced drastically with genotyping and is entirely absent with exome sequencing. [file gb-2014-15-2-r36-S14.png]

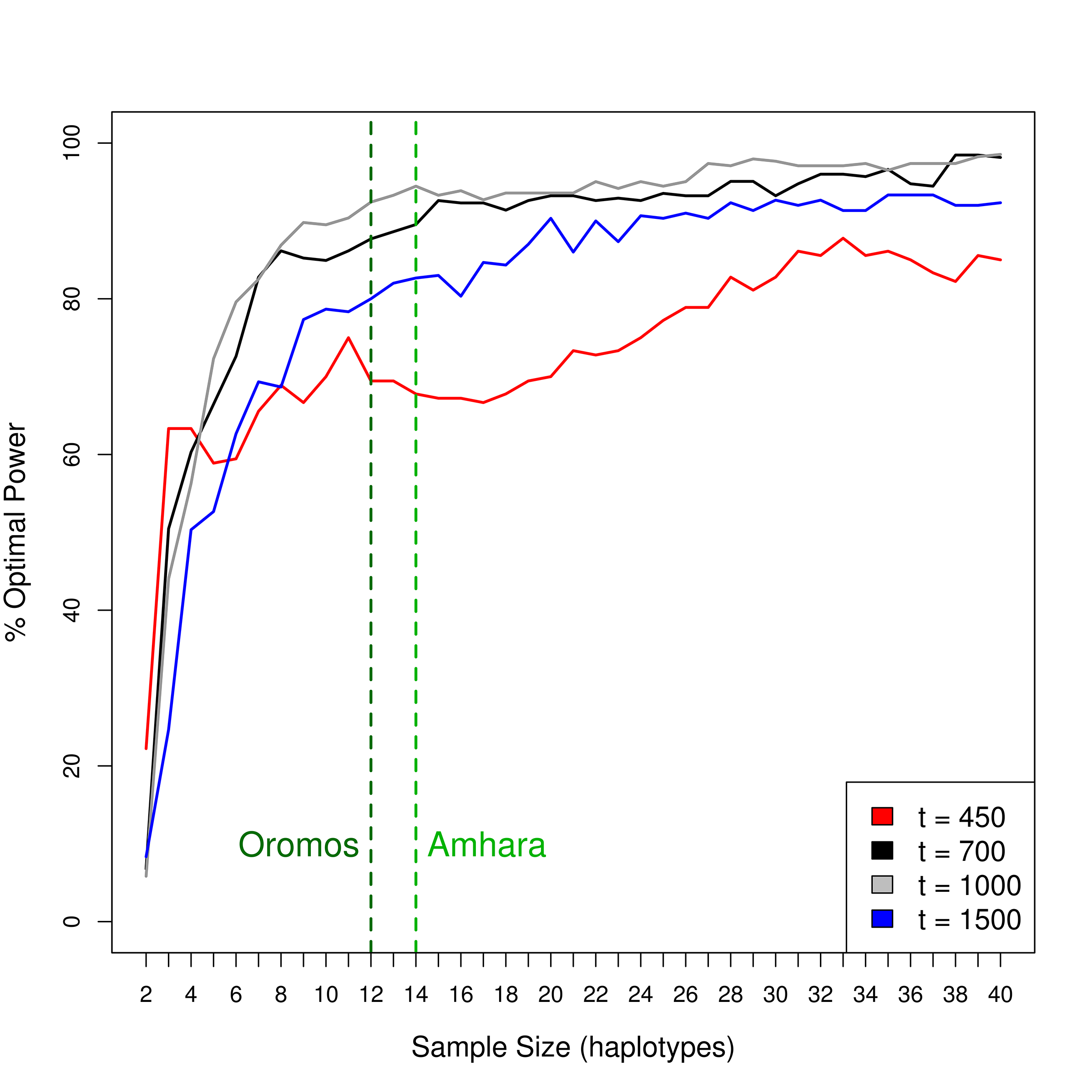

Supplement: Additional file 15: Figure S10 — The impact of sequenced sample size on power, using Sπ as an exemplar test. Five hundred populations were simulated with a fixed selection coefficient of s = 0.02 and sampled at different times after selection start. Sample size is shown in haplotypes, and ranges in n = (2,3,…,40). Optimal power at each time was determined using a large sample size of n = 400. The populations were sampled at four time points representing each of the observed regimes: t = 450 for 'pre-fixation', t = 700 and t = 1,000 for 'near-fixation', and t = 1,500 for 'post-fixation'. Although we see an increase in power as more haploptypes are sampled, sampling 12 or 14 haplotypes (our Oromos and Amhara populations, respectively) yields 67 to 95% of the optimal power. [file gb-2014-15-2-r36-S15.png]
