# Supplementary material for: Whole genome sequencing of Ethiopian highlanders reveals conserved hypoxia tolerance genes
Source: Genome Biol. 2014 Feb 20;15(2):R36. doi: 10.1186/gb-2014-15-2-r36 (PMC4054780; doi:10.1186/gb-2014-15-2-r36)
Supplement: Additional file 6: Table S1 — Regions identified as significant under a 0.1% genome-wide false discovery rate. [file gb-2014-15-2-r36-S6.pdf]

Table S1. Regions identified as significant under a 0.1% genome wide false discovery rate.

Test	Base Pairs (Amhara)	Regions (distinct)	Genes	Base Pairs (Oromos)	Regions (distinct)	Genes
S_f	7,928,000	99	88	7,858,000	95	99
S_π	8,018,000	98	89	8,740,000	114	110
F_{st}	6,824,000	78	71	6,442,000	68	49
PBS	7,670,000	93	79	7,406,000	88	80
**Total**	**23,950,000**	**261**	**272**	**24,744,000**	**260**	**271**
