# Supplementary material for: Whole genome sequencing of Ethiopian highlanders reveals conserved hypoxia tolerance genes
Source: Genome Biol. 2014 Feb 20;15(2):R36. doi: 10.1186/gb-2014-15-2-r36 (PMC4054780; doi:10.1186/gb-2014-15-2-r36)
Supplement: Additional file 9 — Test statistic values on chromosomes 1 to 22, as well as X, in the Amhara and the Oromos populations. The tests shown are PBS, Fst, Sπ, and Sf. Regions exceeding the 0.1% genomic FDR and that passed all prioritization criteria are shown in green. [file gb-2014-15-2-r36-S9.pdf]

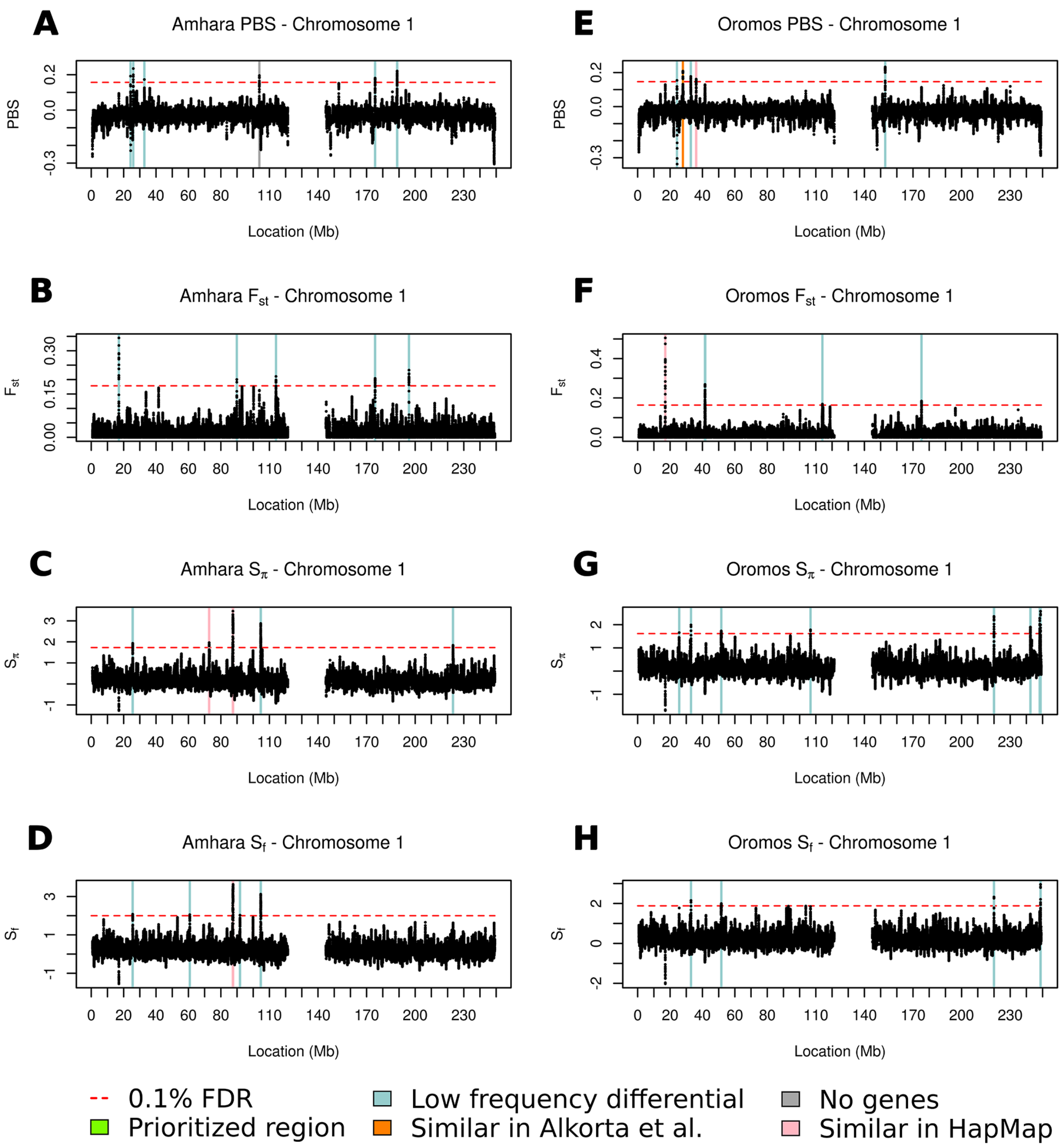

Chromosome 1. Test statistic values in the Amhara (A-D) and Oromos (E-H) populations. The tests shown are PBS (A, E), F_{st} (B, F), S_π (C, G), and S_f (D, H). The four filters that were used to shortlist the regions are color-coded. Regions above the 0.1% FDR that passed all filters are shown in green.

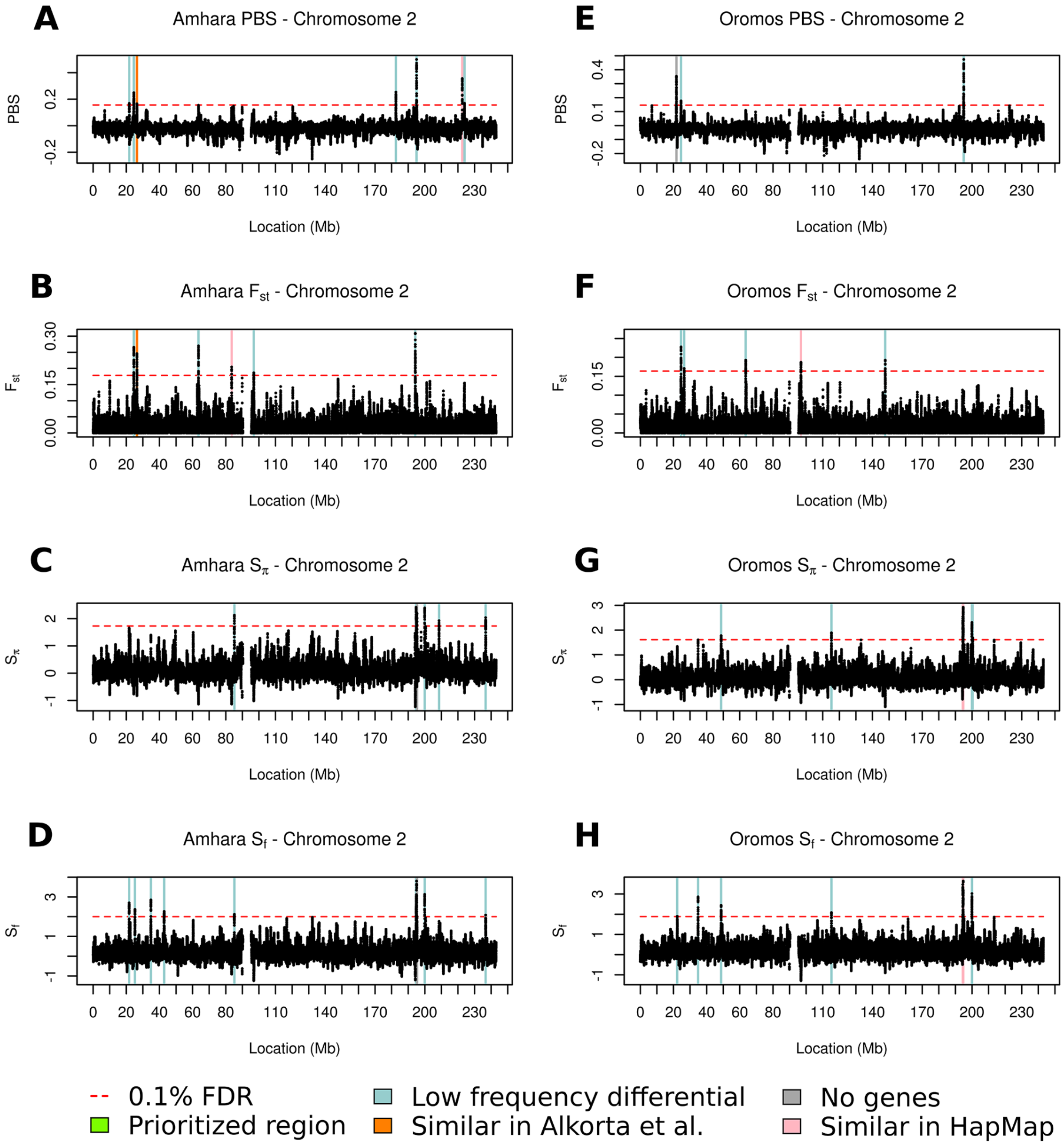

Chromosome 2. Test statistic values in the Amhara (A-D) and Oromos (E-H) populations. The tests shown are PBS (A, E), F_{st} (B, F), S_π (C, G), and S_f (D, H). The four prioritization filters that were used to shortlist the regions are color-coded. Regions above the 0.1% FDR that passed all filters are shown in green.

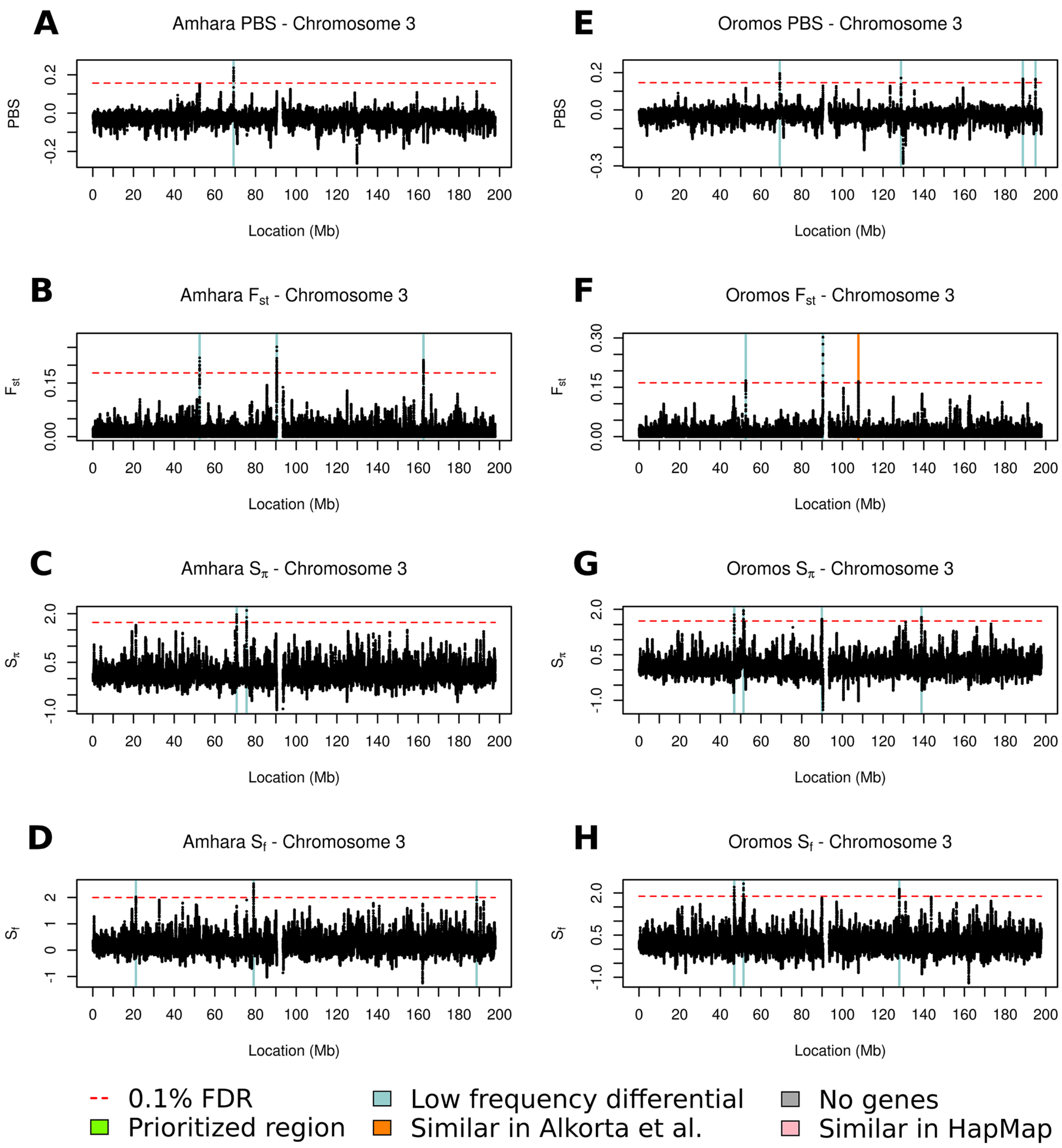

Chromosome 3. Test statistic values in the Amhara (A-D) and Oromos (E-H) populations. The tests shown are PBS (A, E), F_{st} (B, F), S_π (C, G), and S_f (D, H). The four prioritization filters that were used to shortlist the regions are color-coded. Regions above the 0.1% FDR that passed all filters are shown in green.

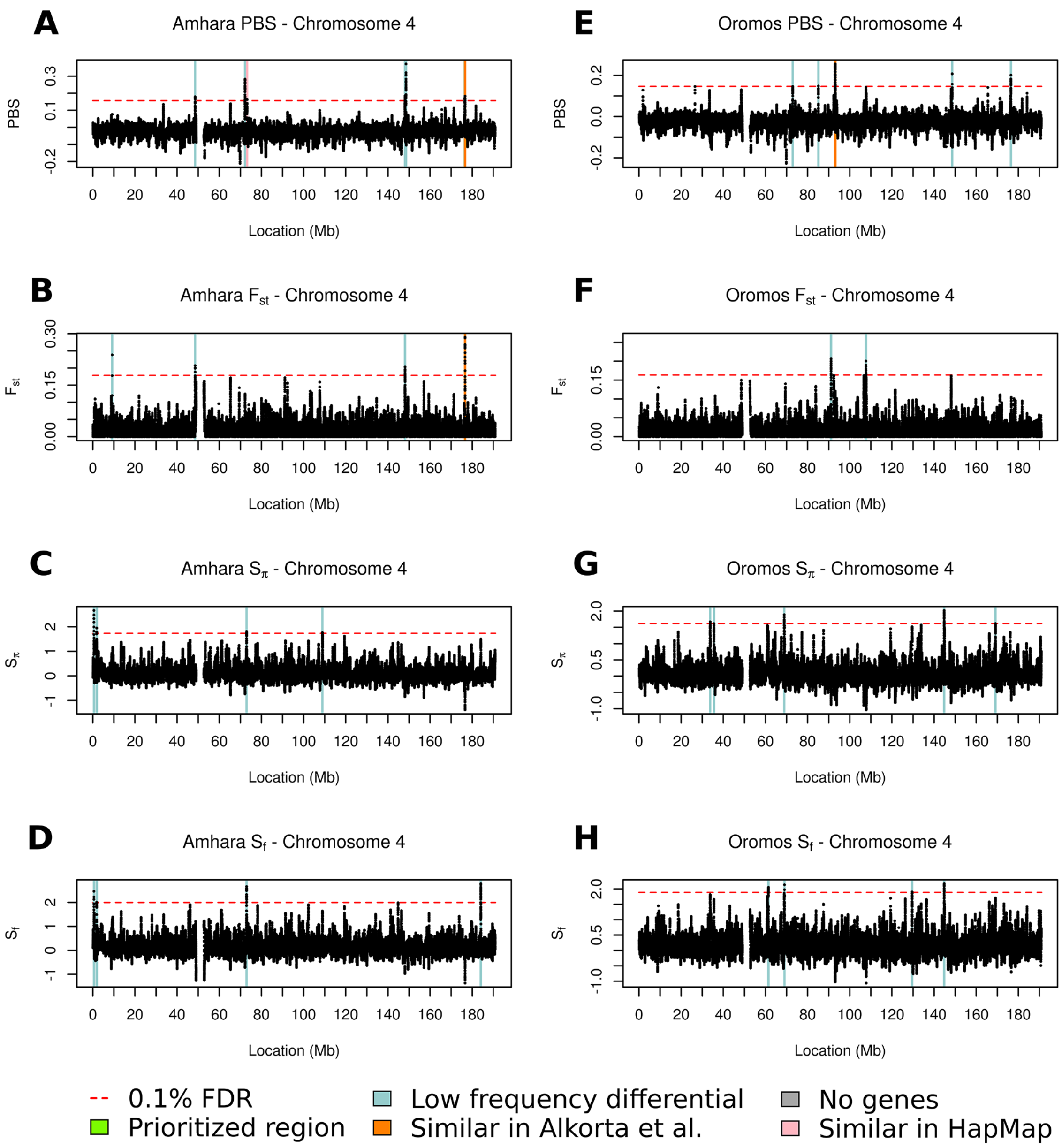

Chromosome 4. Test statistic values in the Amhara (A-D) and Oromos (E-H) populations. The tests shown are PBS (A, E), F_{st} (B, F), S_π (C, G), and S_f (D, H). The four prioritization filters that were used to shortlist the regions are color-coded. Regions above the 0.1% FDR that passed all filters are shown in green.

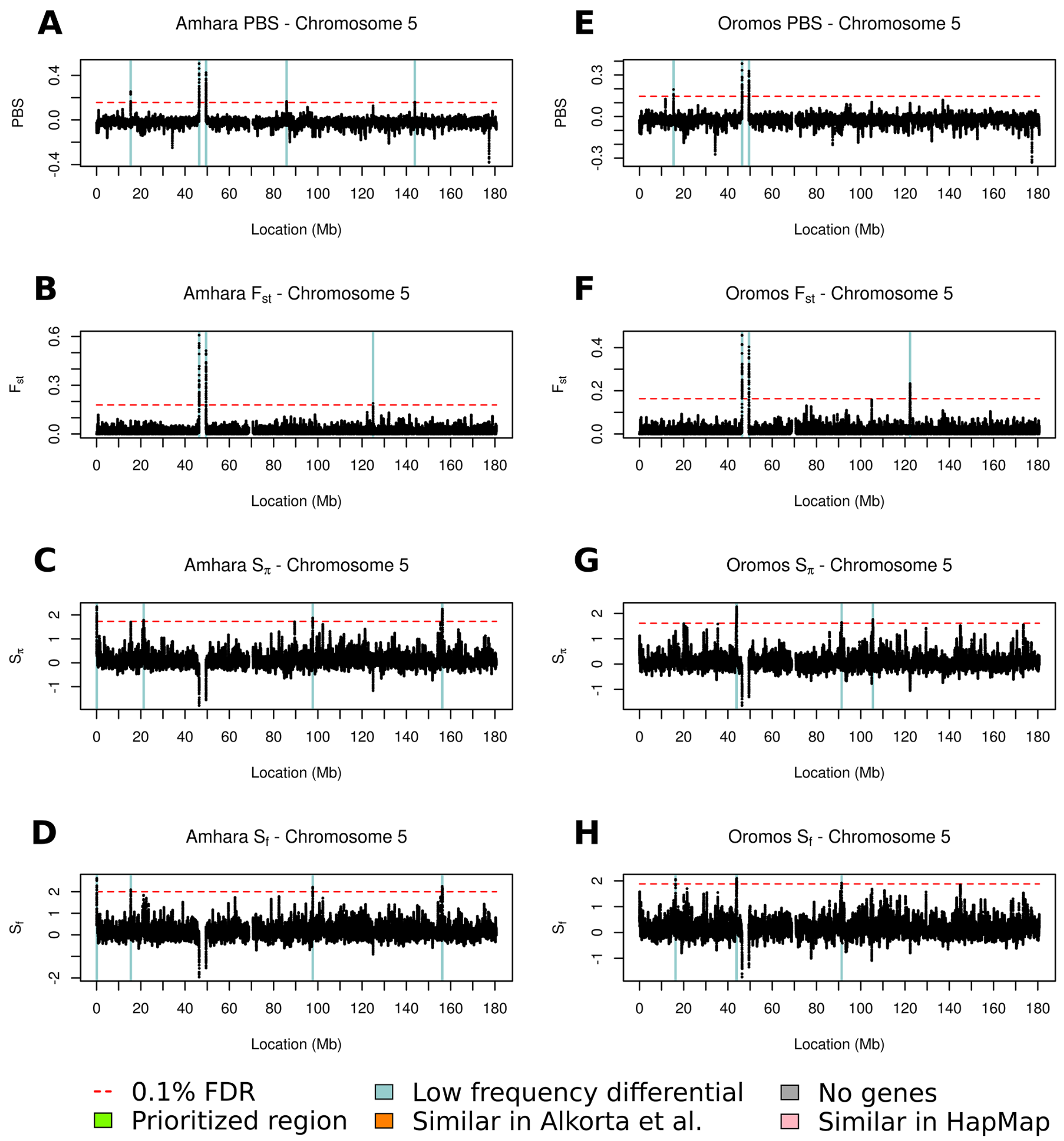

Chromosome 5. Test statistic values in the Amhara (A-D) and Oromos (E-H) populations. The tests shown are PBS (A, E), F_{st} (B, F), S_π (C, G), and S_f (D, H). The four prioritization filters that were used to shortlist the regions are color-coded. Regions above the 0.1% FDR that passed all filters are shown in green.

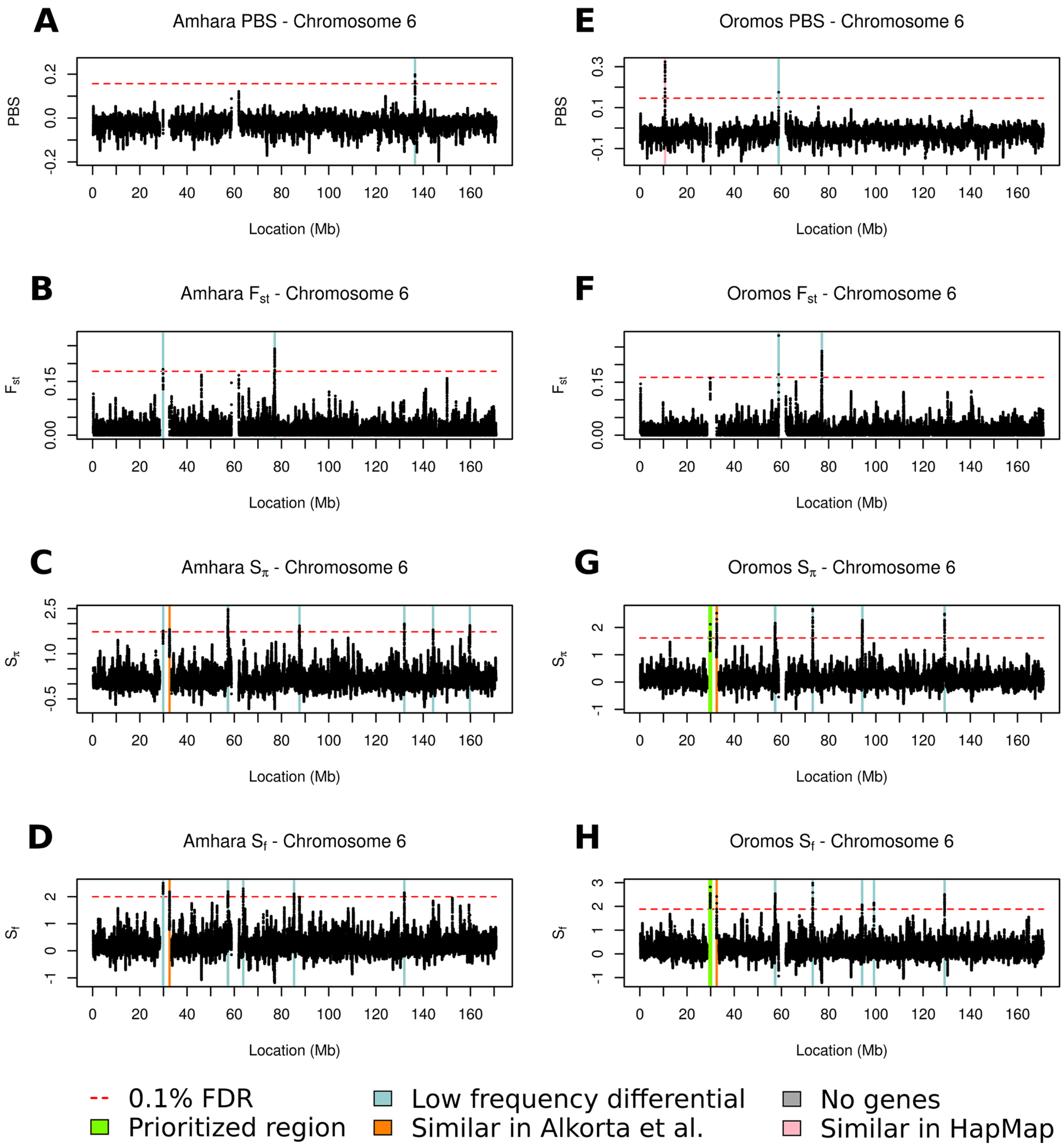

Chromosome 6. Test statistic values in the Amhara (A-D) and Oromos (E-H) populations. The tests shown are PBS (A, E), F_{st} (B, F), S_π (C, G), and S_f (D, H). The four prioritization filters that were used to shortlist the regions are color-coded. Regions above the 0.1% FDR that passed all filters are shown in green. Specifically, the green region in the Oromos vs. LWK S_π and S_f statistics, position 29.8M, contains four genes (*HLA-G, HLA-H, HCG2P7*, and *HCG4P6*) and has S_π and S_f values of 2.12 and 2.82, respectively.

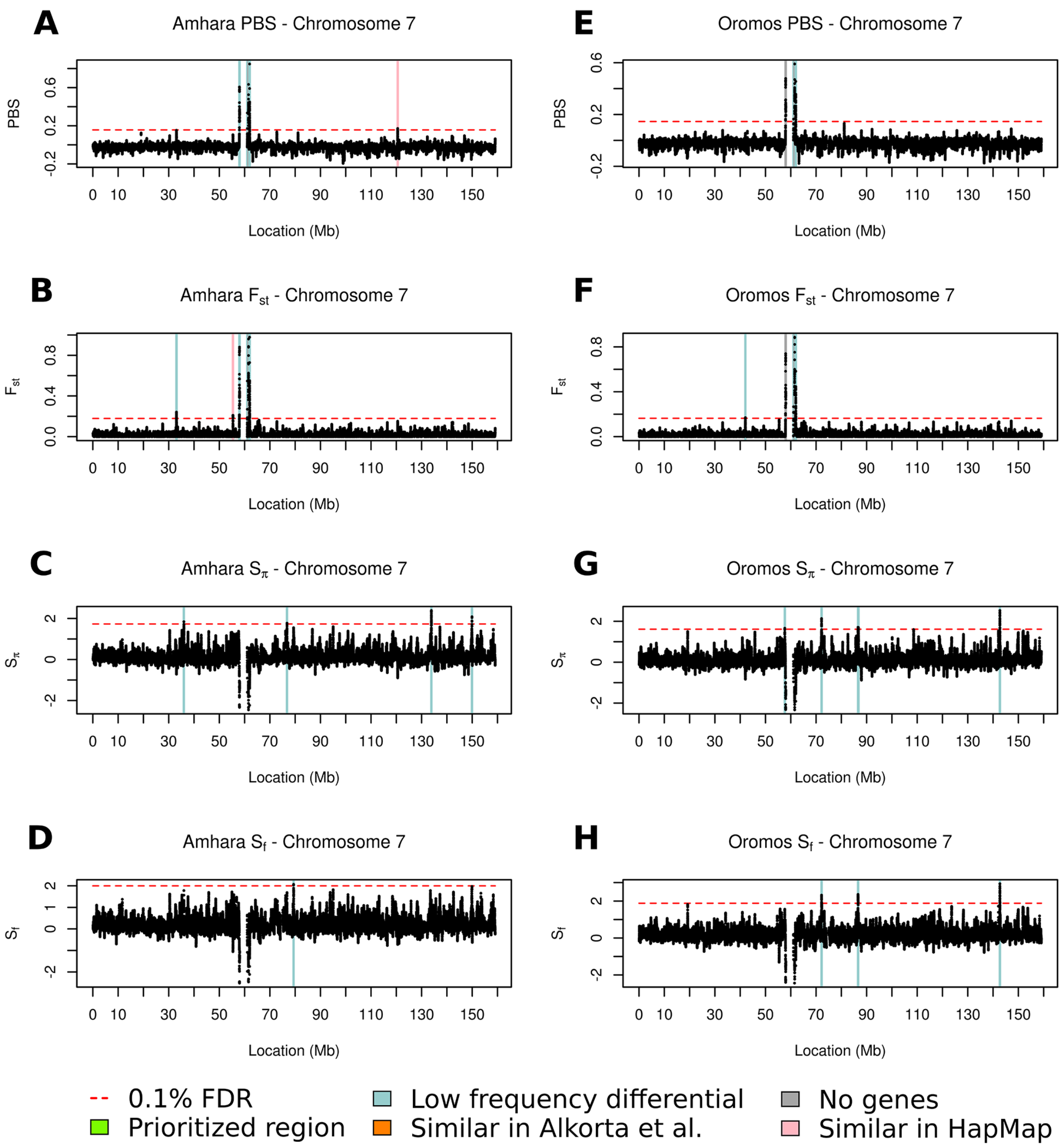

Chromosome 7. Test statistic values in the Amhara (A-D) and Oromos (E-H) populations. The tests shown are PBS (A, E), F_{st} (B, F), S_{π} (C, G), and S_f (D, H). The four prioritization filters that were used to shortlist the regions are color-coded. Regions above the 0.1% FDR that passed all filters are shown in green.

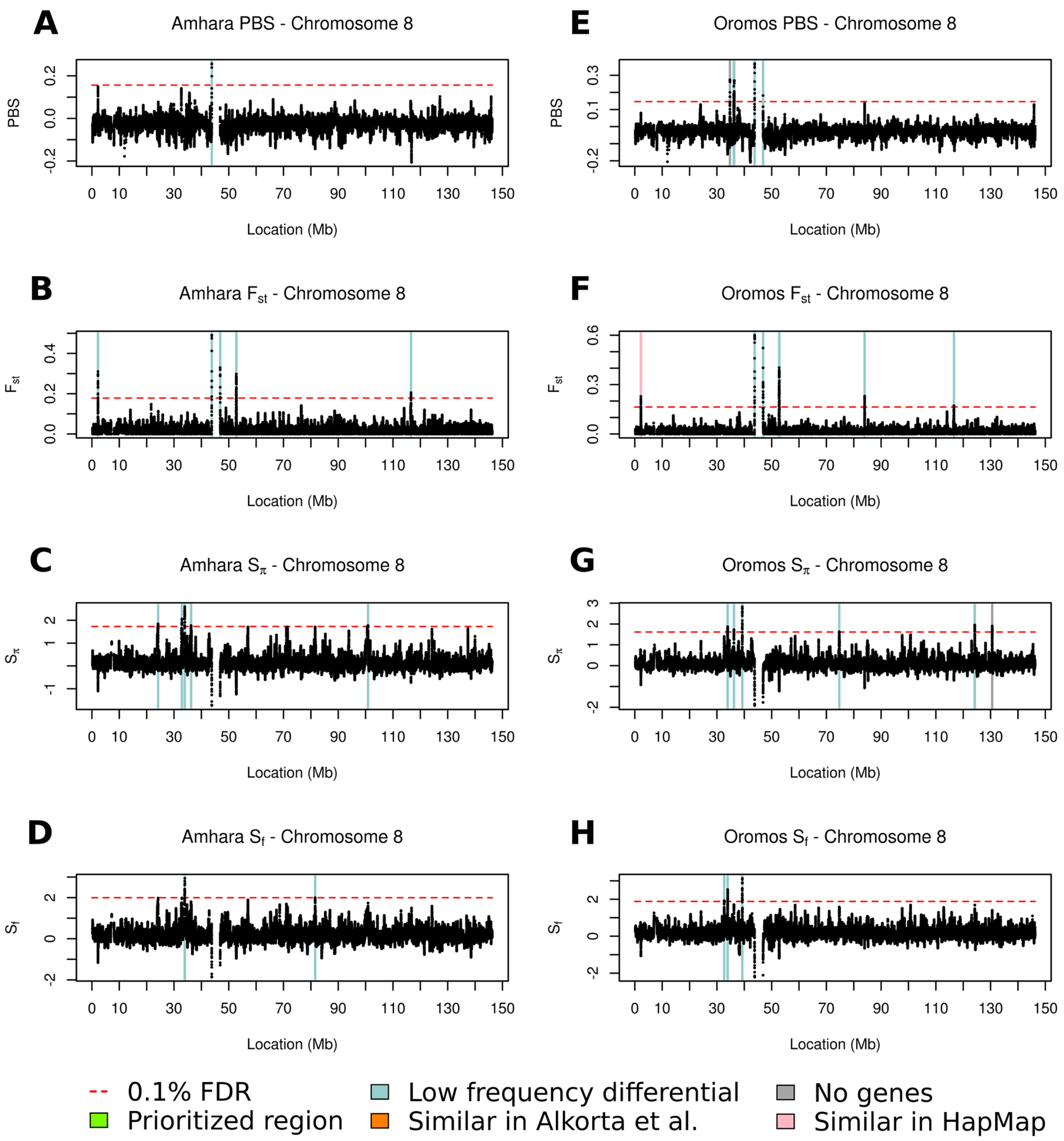

Chromosome 8. Test statistic values in the Amhara (A-D) and Oromos (E-H) populations. The tests shown are PBS (A, E), F_{st} (B, F), S_π (C, G), and S_f (D, H). The four prioritization filters that were used to shortlist the regions are color-coded. Regions above the 0.1% FDR that passed all filters are shown in green.

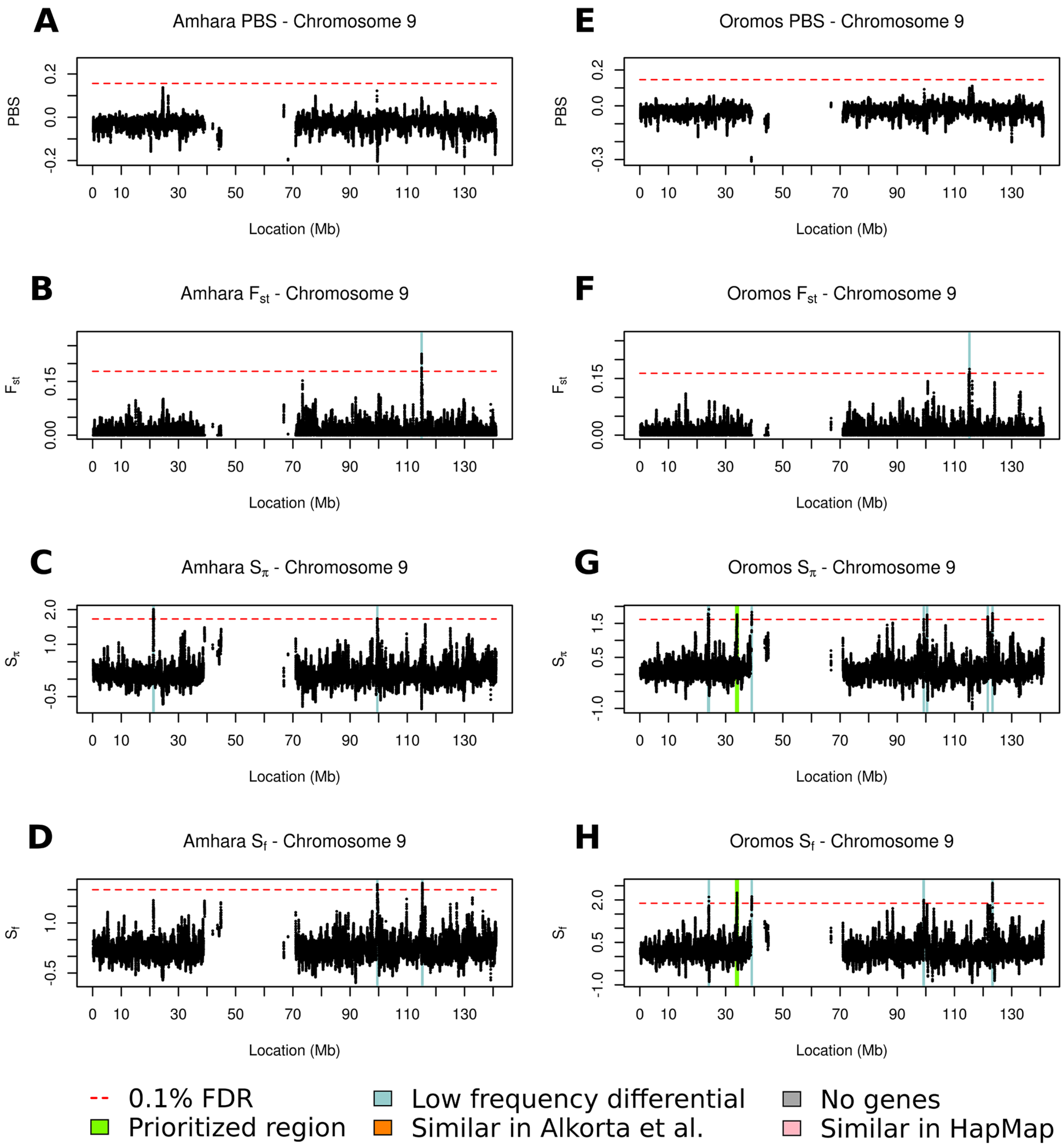

Chromosome 9. Test statistic values in the Amhara (A-D) and Oromos (E-H) populations. The tests shown are PBS (A, E), F_{st} (B, F), S_π (C, G), and S_f (D, H). The four prioritization filters that were used to shortlist the regions are color-coded. Regions above the 0.1% FDR that passed all filters are shown in green. Specifically, the green region in the Oromos vs. LWK S_π and S_f statistics, position 34M, contains four genes (*UBE2R2, UBAP2, SNORD121B*, and *SNORD121A*) and has S_π and S_f values of 1.76 and 2.25, respectively.

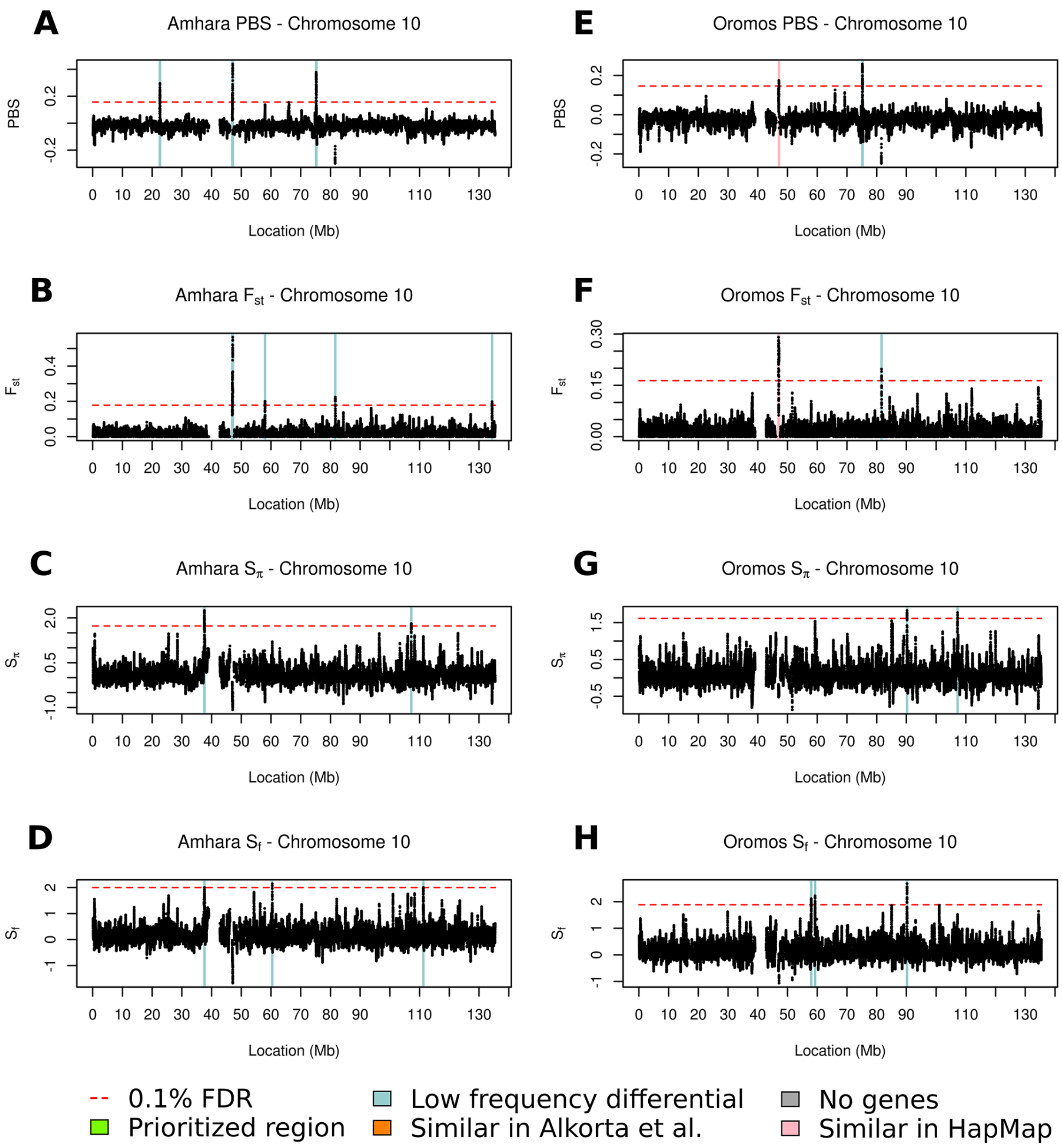

Chromosome 10. Test statistic values in the Amhara (A-D) and Oromos (E-H) populations. The tests shown are PBS (A, E), F_{st} (B, F), S_{π} (C, G), and S_f (D, H). The four prioritization filters that were used to shortlist the regions are color-coded. Regions above the 0.1% FDR that passed all filters are shown in green.

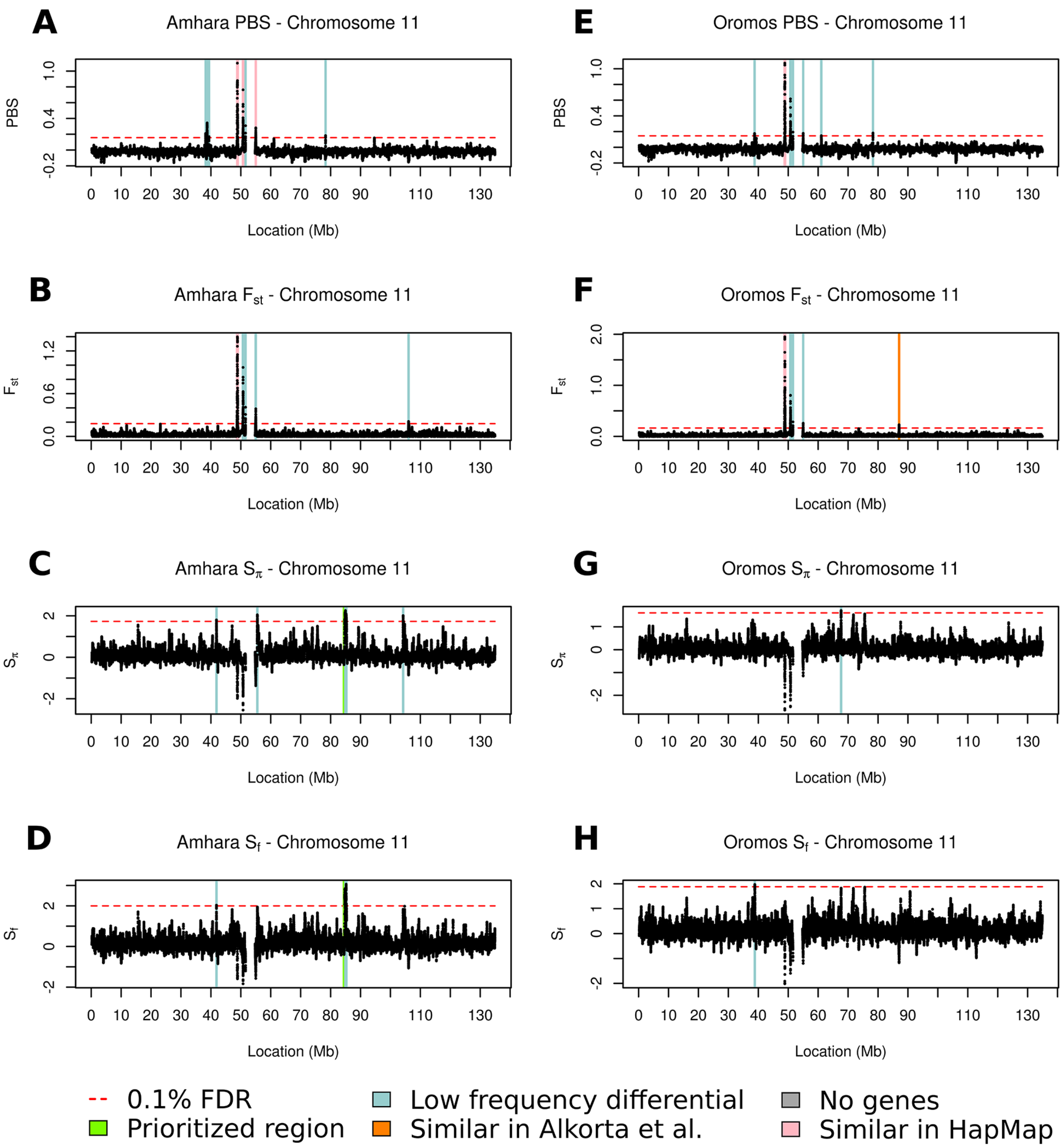

Chromosome 11. Test statistic values in the Amhara (A-D) and Oromos (E-H) populations. The tests shown are PBS (A, E), F_{st} (B, F), S_π (C, G), and S_f (D, H). The four prioritization filters that were used to shortlist the regions are color-coded. Regions above the 0.1% FDR that passed all filters are shown in green. Specifically, the green region in the Amhara vs. LWK S_π and S_f statistics, position 84.7M, contains one gene (*DLG2*) and has S_π and S_f values of 2.16 and 2.85, respectively.

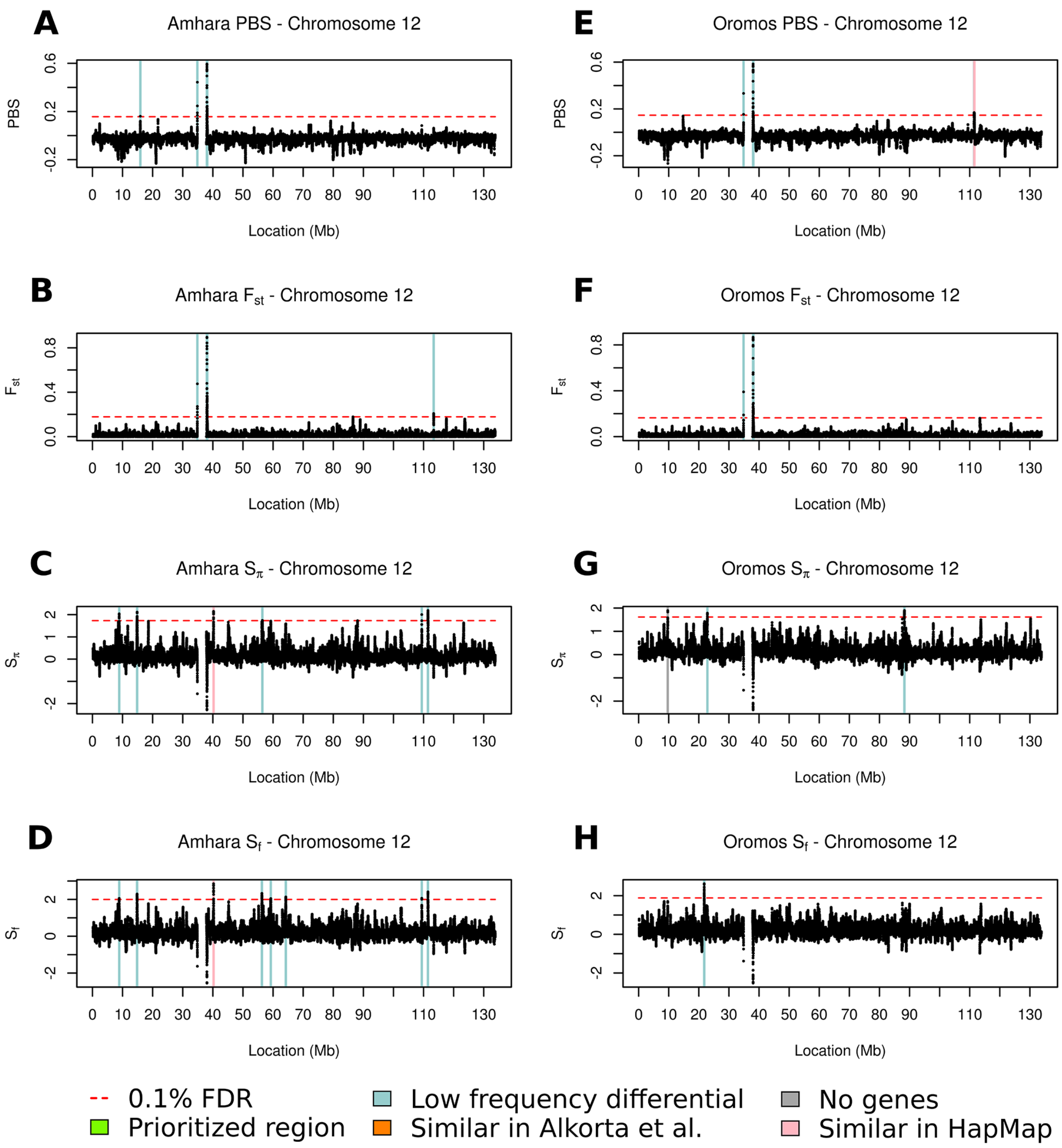

Chromosome 12. Test statistic values in the Amhara (A-D) and Oromos (E-H) populations. The tests shown are PBS (A, E), F_{st} (B, F), S_π (C, G), and S_f (D, H). The four prioritization filters that were used to shortlist the regions are color-coded. Regions above the 0.1% FDR that passed all filters are shown in green.

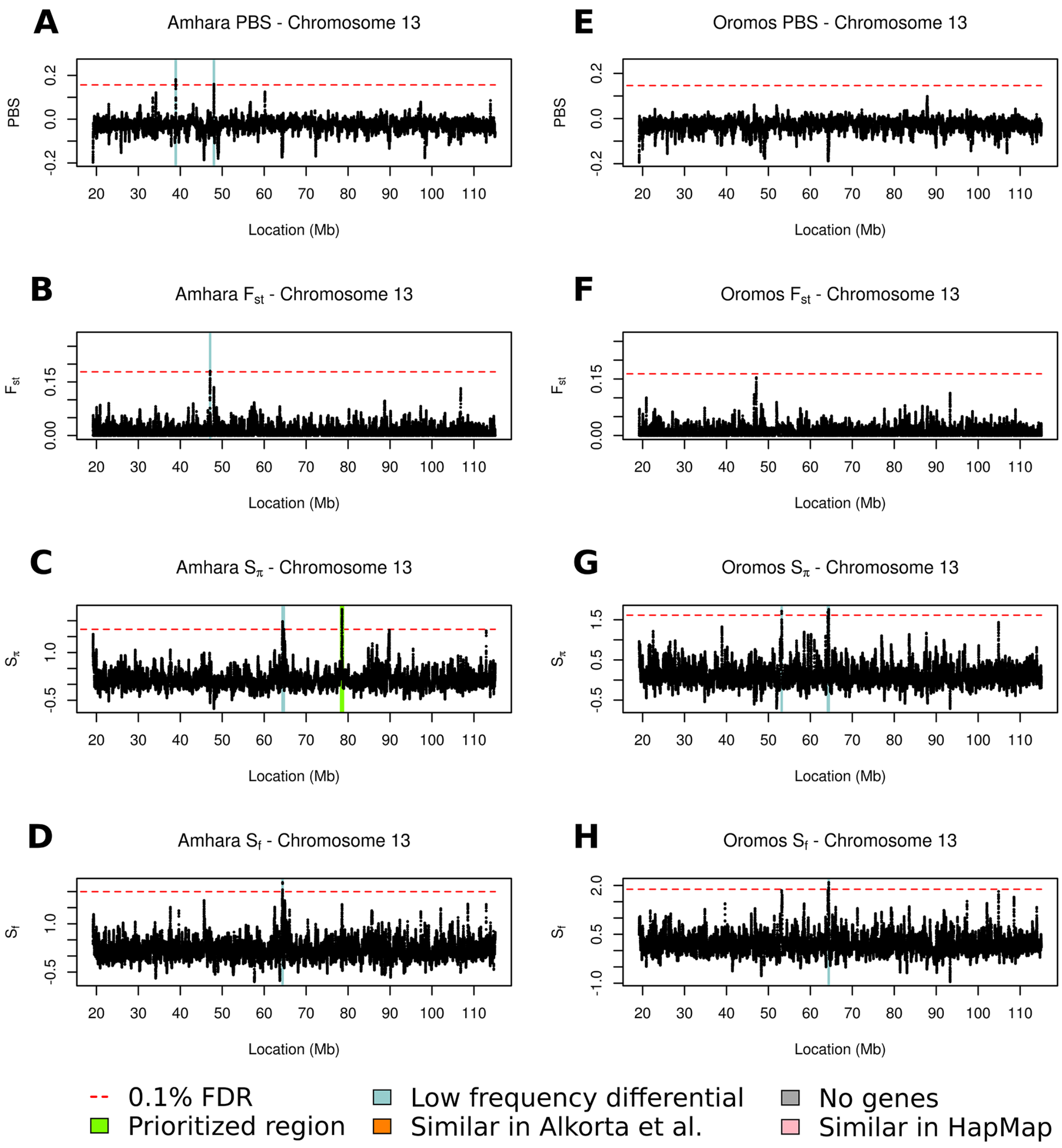

Chromosome 13. Test statistic values in the Amhara (A-D) and Oromos (E-H) populations. The tests shown are PBS (A, E), F_{st} (B, F), S_π (C, G), and S_f (D, H). The four prioritization filters that were used to shortlist the regions are color-coded. Regions above the 0.1% FDR that passed all filters are shown in green. Specifically, the green region in the Amhara vs. LWK S_π statistic, position 78.5M, contains one gene (*EDNRB*), and has S_π statistic value of 2.36.

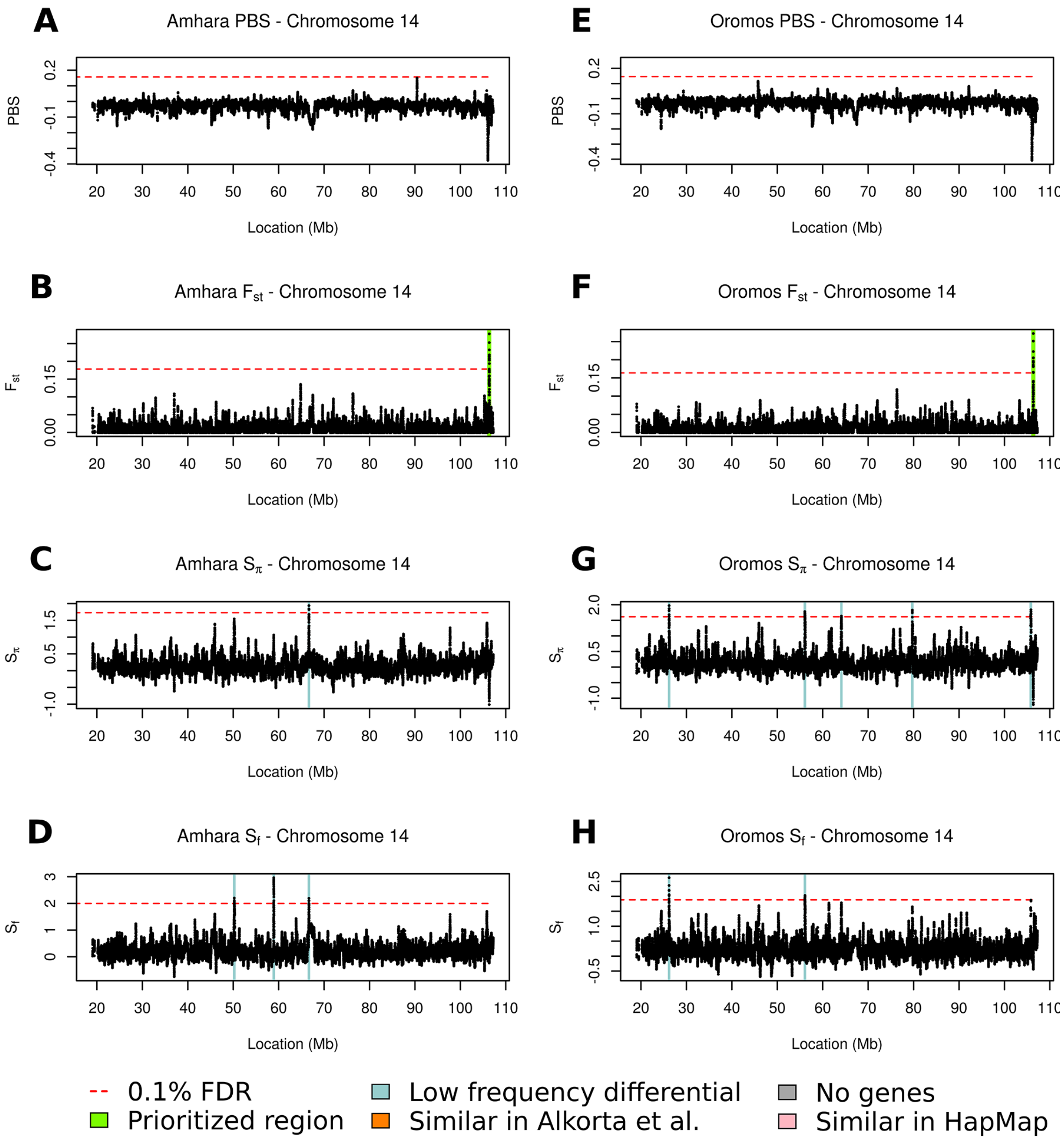

Chromosome 14. Test statistic values in the Amhara (A-D) and Oromos (E-H) populations. The tests shown are PBS (A, E), F_{st} (B, F), S_{π} (C, G), and S_f (D, H). The four prioritization filters that were used to shortlist the regions are color-coded. Regions above the 0.1% FDR that passed all filters are shown in green. Specifically, the green region in the Amhara vs. LWK and Oromos vs. LWK F_{st} statistic, position 106.3M, contains one gene (*KIAA0125*), and has F_{st} statistic values of 0.28 and 0.27, respectively.

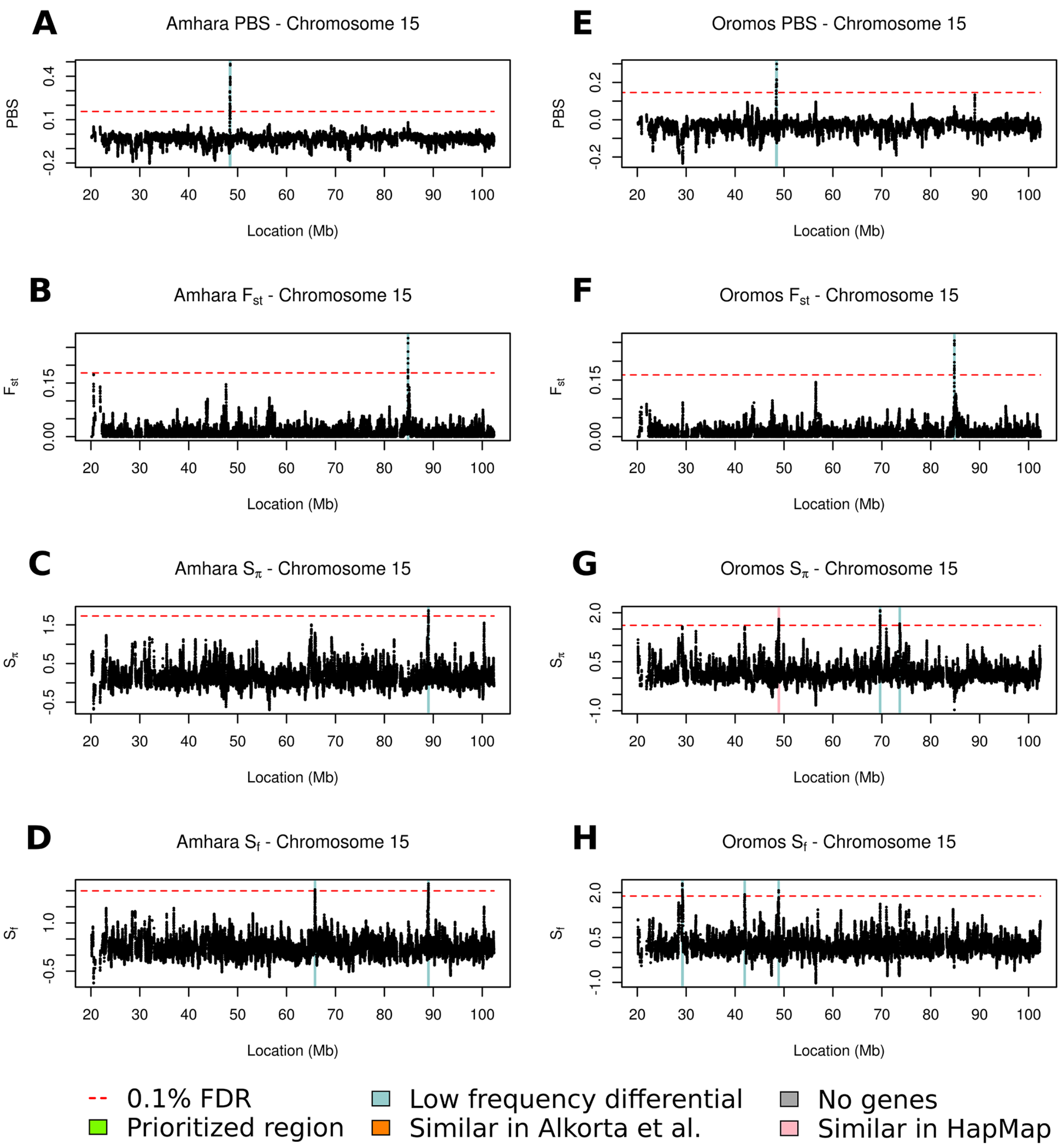

Chromosome 15. Test statistic values in the Amhara (A-D) and Oromos (E-H) populations. The tests shown are PBS (A, E), F_{st} (B, F), S_{π} (C, G), and S_f (D, H). The four prioritization filters that were used to shortlist the regions are color-coded. Regions above the 0.1% FDR that passed all filters are shown in green.

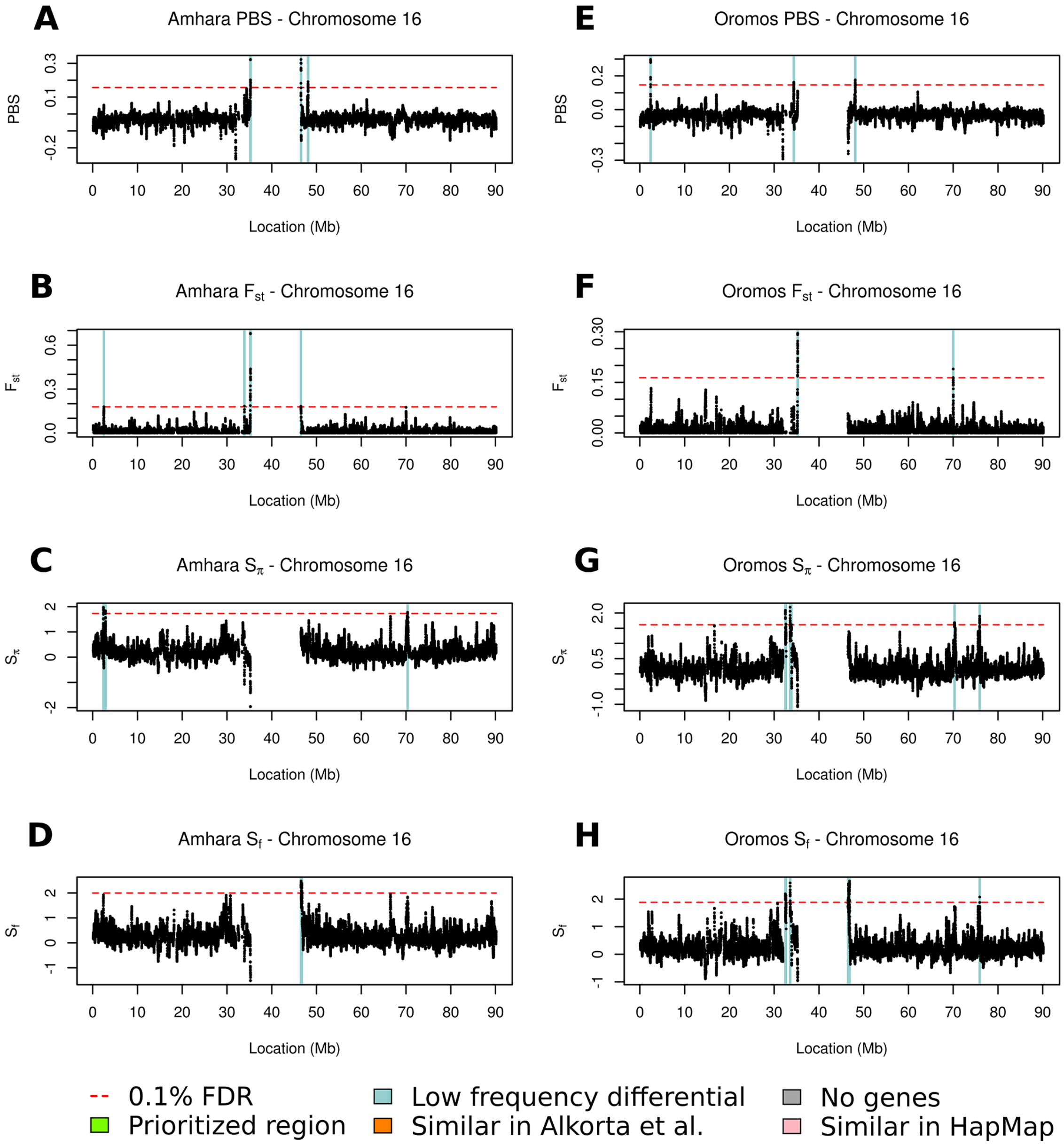

Chromosome 16. Test statistic values in the Amhara (A-D) and Oromos (E-H) populations. The tests shown are PBS (A, E), F_{st} (B, F), S_π (C, G), and S_f (D, H). The four prioritization filters that were used to shortlist the regions are color-coded. Regions above the 0.1% FDR that passed all filters are shown in green.

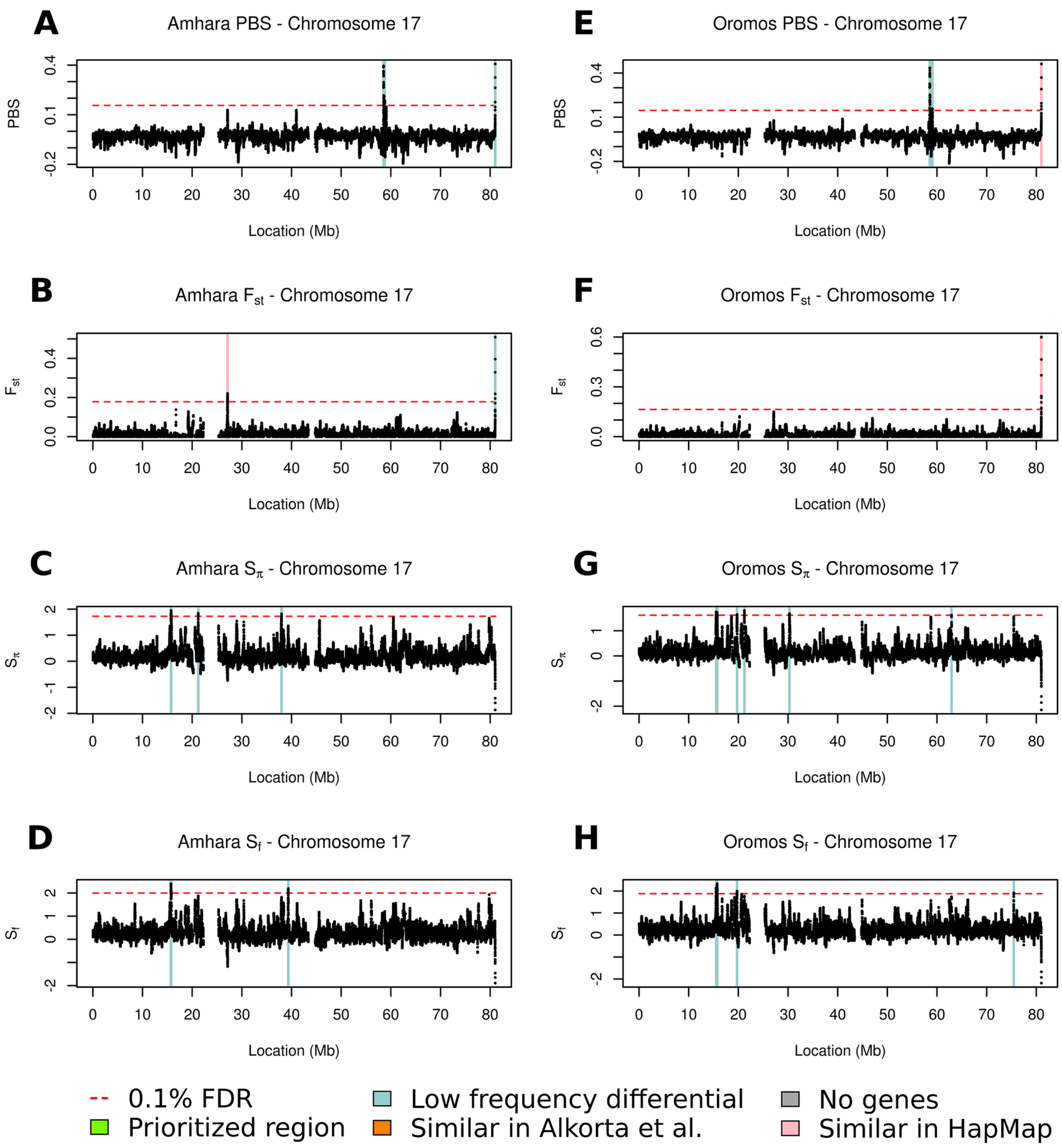

Chromosome 17. Test statistic values in the Amhara (A-D) and Oromos (E-H) populations. The tests shown are PBS (A, E), F_{st} (B, F), S_π (C, G), and S_f (D, H). The four prioritization filters that were used to shortlist the regions are color-coded. Regions above the 0.1% FDR that passed all filters are shown in green.

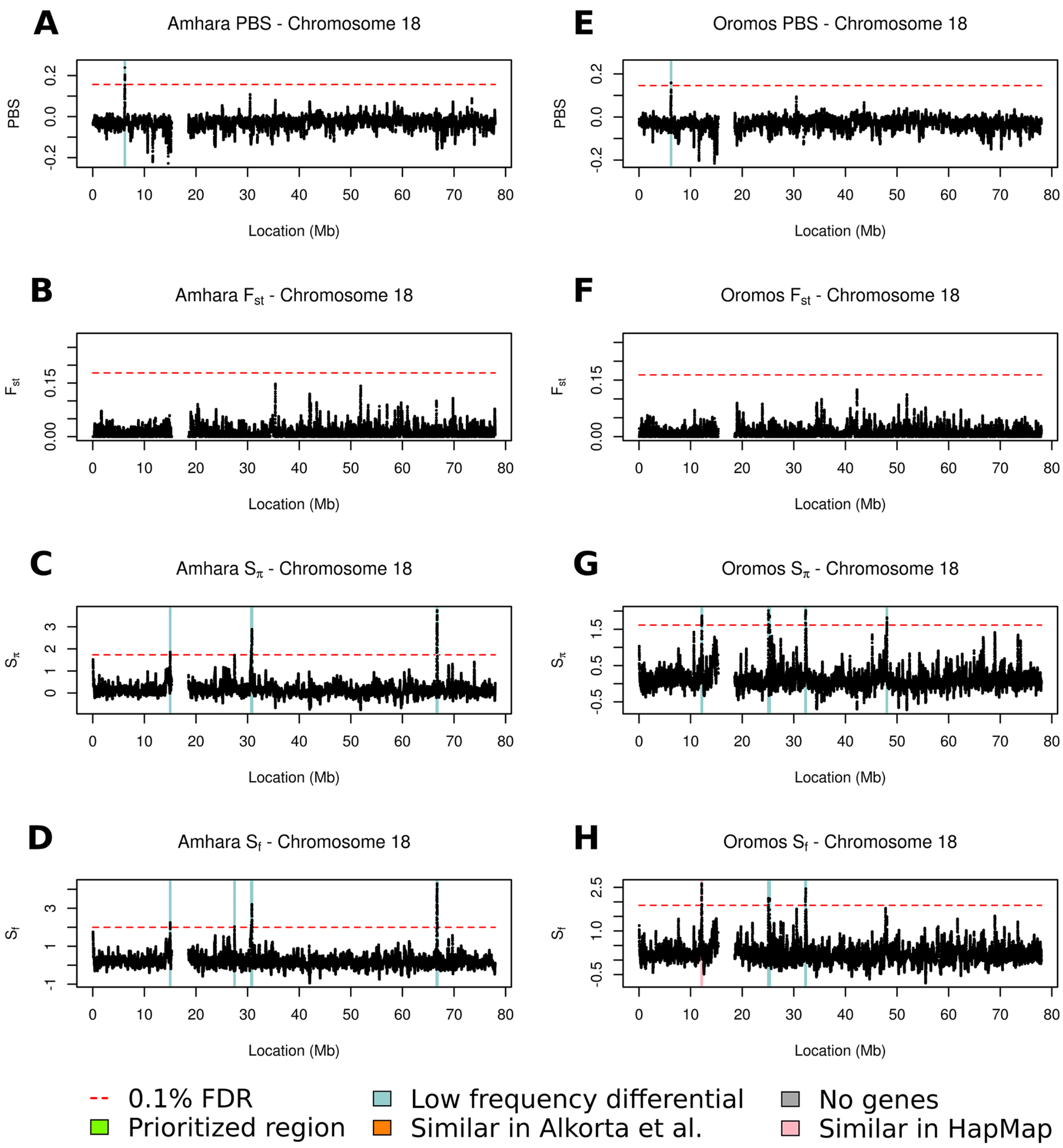

Chromosome 18. Test statistic values in the Amhara (A-D) and Oromos (E-H) populations. The tests shown are PBS (A, E), F_{st} (B, F), S_{π} (C, G), and S_f (D, H). The four prioritization filters that were used to shortlist the regions are color-coded. Regions above the 0.1% FDR that passed all filters are shown in green.

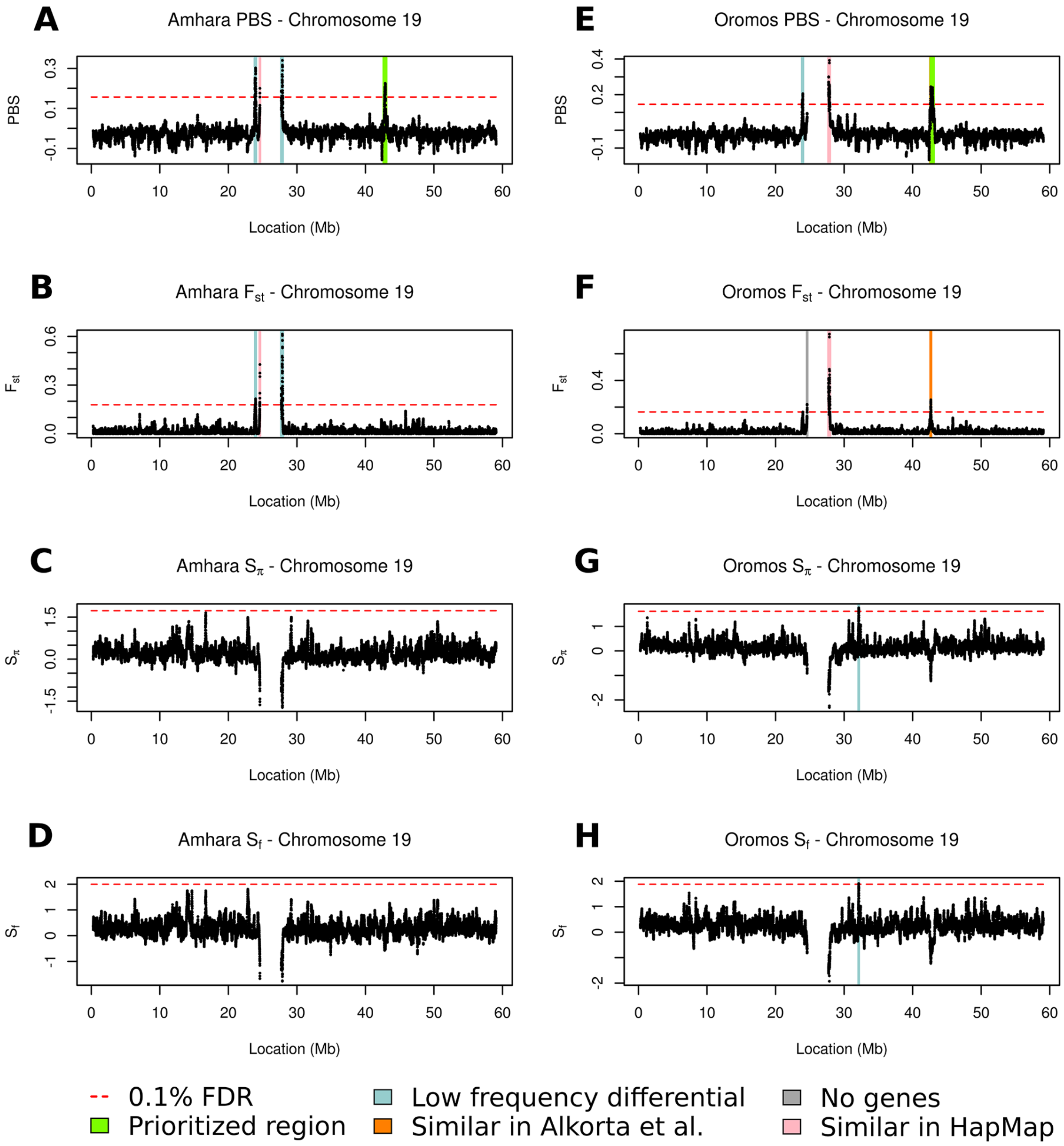

Chromosome 19. Test statistic values in the Amhara (A-D) and Oromos (E-H) populations. The tests shown are *PBS* (A, E), F_{st} (B, F), S_π (C, G), and S_f (D, H). The four prioritization filters that were used to shortlist the regions are color-coded. Regions above the 0.1% FDR that passed all filters are shown in green. Specifically, the green region in both the Amhara vs. LWK and Oromos vs. LWK *PBS* statistic, position 42.74-42.97M, contains ten genes (*GSK3A*, *ERF*, *CIC*, *PAFAH1B3*, *PRR19*, *TMEM145*, *MEGF8*, *CNFN*, *LIPE*, and *CXCL17*), and has *PBS* statistic values of 0.226 and 0.24, respectively.

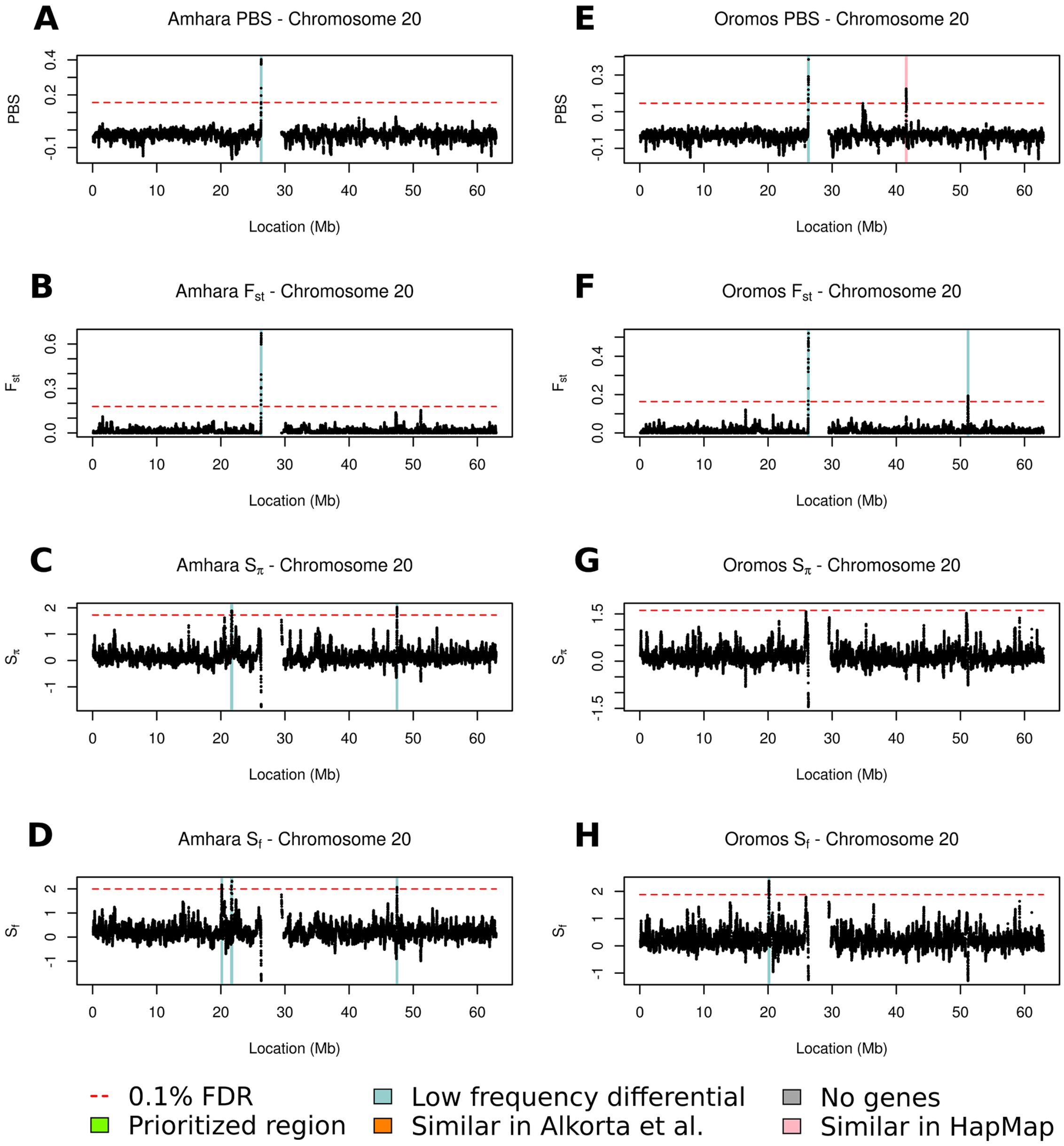

Chromosome 20. Test statistic values in the Amhara (A-D) and Oromos (E-H) populations. The tests shown are PBS (A, E), F_{st} (B, F), S_π (C, G), and S_f (D, H). The four prioritization filters that were used to shortlist the regions are color-coded. Regions above the 0.1% FDR that passed all filters are shown in green.

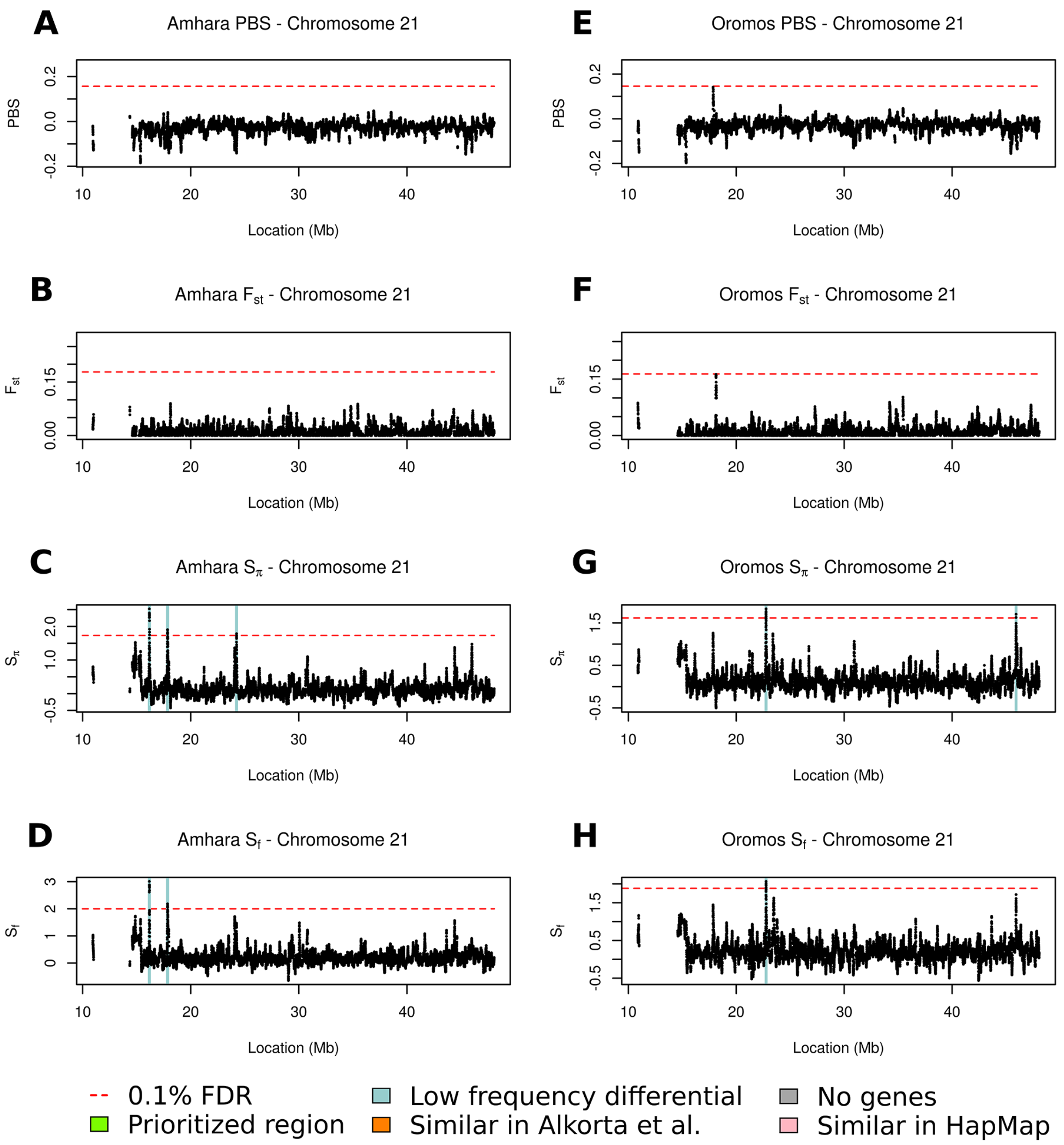

Chromosome 21. Test statistic values in the Amhara (A-D) and Oromos (E-H) populations. The tests shown are PBS (A, E), F_{st} (B, F), S_π (C, G), and S_f (D, H). The four prioritization filters that were used to shortlist the regions are color-coded. Regions above the 0.1% FDR that passed all filters are shown in green.

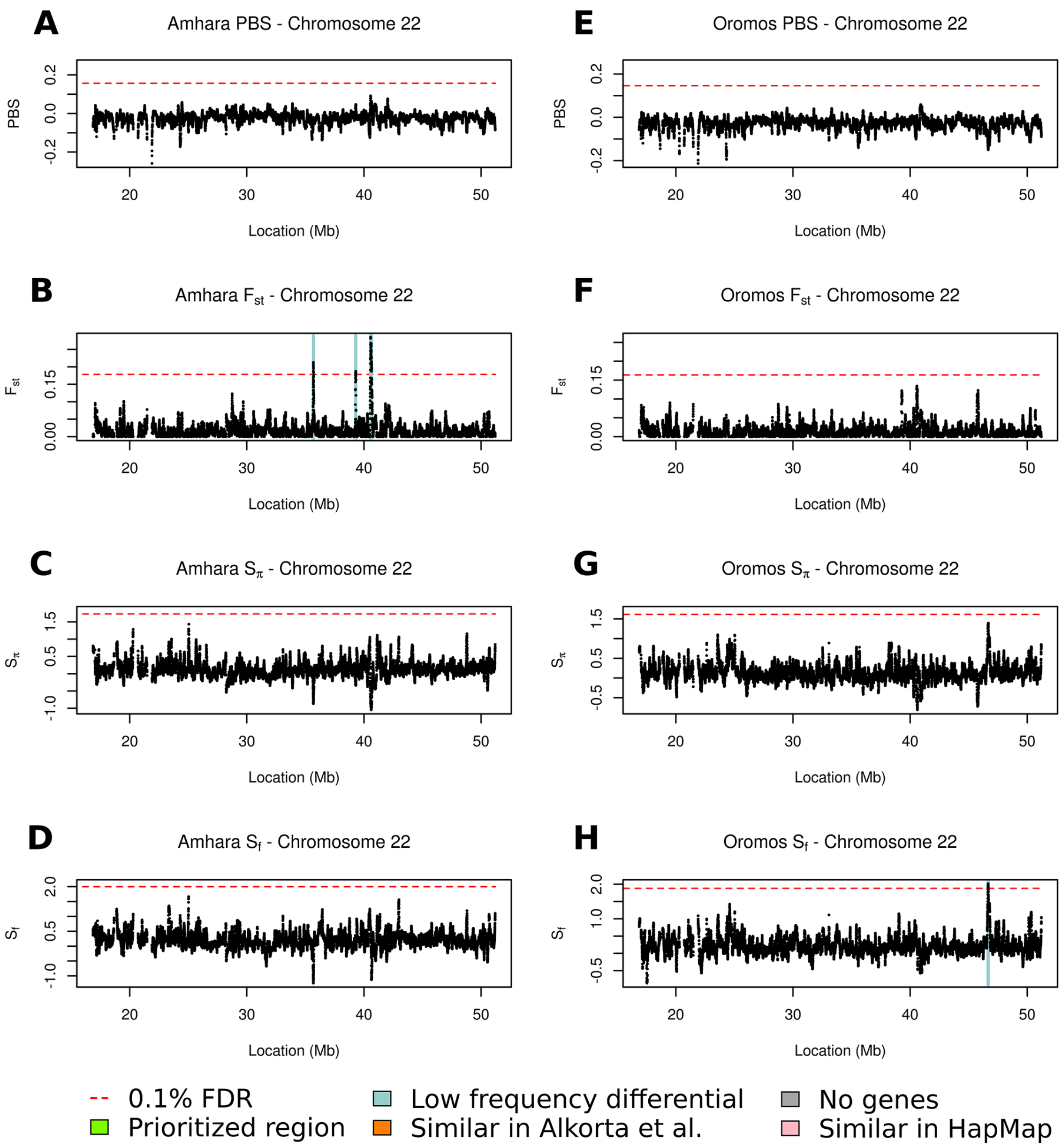

Chromosome 22. Test statistic values in the Amhara (A-D) and Oromos (E-H) populations. The tests shown are PBS (A, E), F_{st} (B, F), S_π (C, G), and S_f (D, H). The four prioritization filters that were used to shortlist the regions are color-coded. Regions above the 0.1% FDR that passed all filters are shown in green.

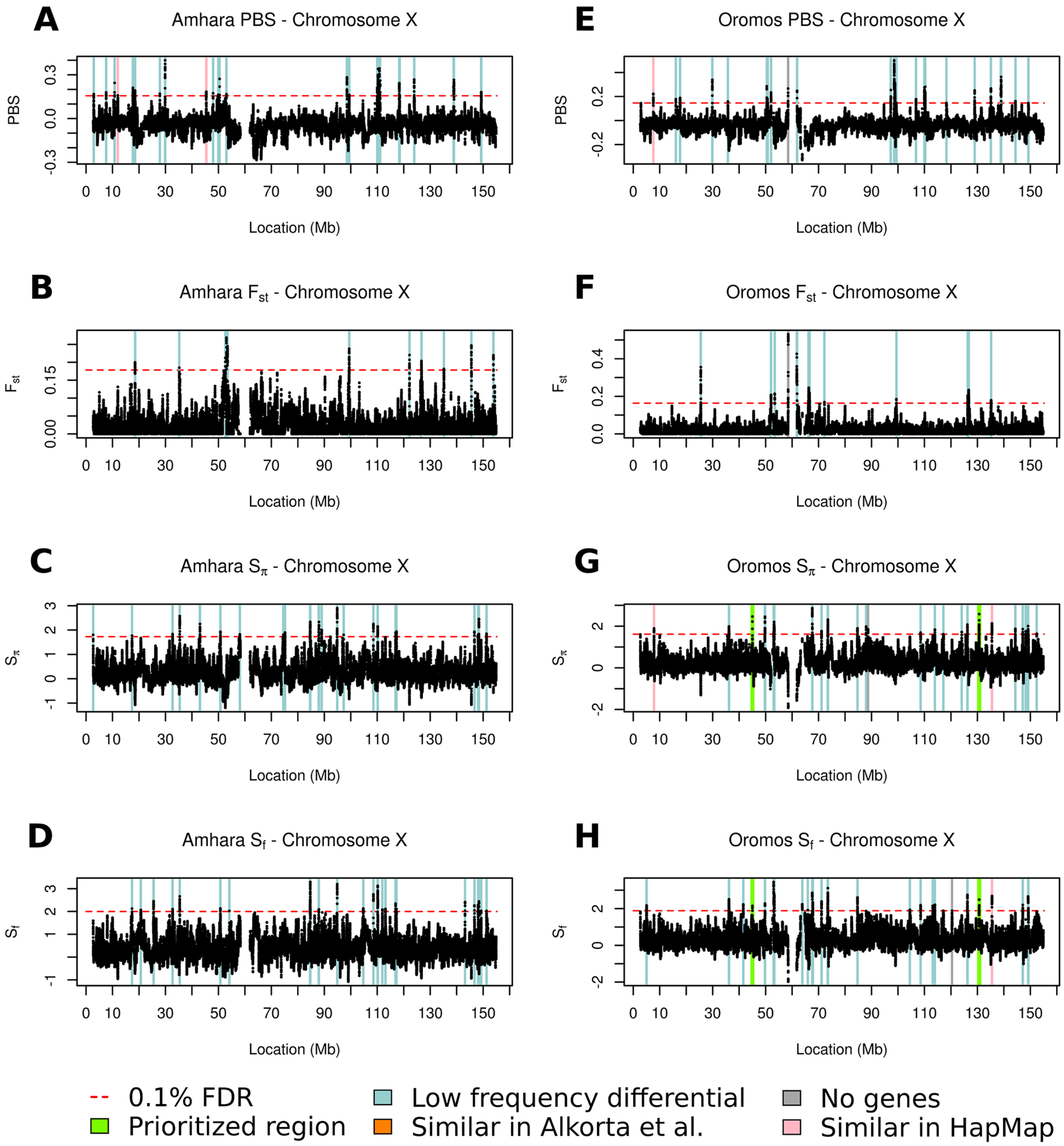

Chromosome X. Test statistic values in the Amhara (A-D) and Oromos (E-H) populations. The tests shown are PBS (A, E), F_{st} (B, F), S_π (C, G), and S_f (D, H). The four filters that were used to shortlist the regions are color-coded. Regions above the 0.1% FDR that passed all filters are shown in green. Specifically, the (left) green region in the Oromos vs. LWK S_π and S_f statistics, position 45.0M, contains one gene (*CXorf36*), and has S_π and S_f statistic values of 2.46 and 2.15, respectively. In addition, the (right) green region in the Oromos vs. LWK S_π and S_f statistic, position 130M, contains one gene (*OR13H1*), and has S_π and S_f statistic value of 2.58 and 2.49, respectively.
