# Supplementary material for: Whole genome sequencing of Ethiopian highlanders reveals conserved hypoxia tolerance genes
Source: Genome Biol. 2014 Feb 20;15(2):R36. doi: 10.1186/gb-2014-15-2-r36 (PMC4054780; doi:10.1186/gb-2014-15-2-r36)
Supplement: Additional file 11: Table S2 — Nonsynonymous SNPs with significant frequency differential in one of the eight prioritized regions. [file gb-2014-15-2-r36-S11.pdf]

Table S2. Nonsynonymous SNPs with significant frequency differential in one of the eight prioratized regions.

Chr	Position	dbSNP	Gene	Transcript	AA Mutation	SIFT P-val
chr9	34017106	rs1785506	UBAP2	NM_018449	R14Q	0.2
chr19	42906914	rs7246232	LIPE	NM_005357	R938S	0.53
chr19	42931004	rs16975750	LIPE	NM_005357	Y100H	0.54
