# Supplementary material for: Whole genome sequencing of Ethiopian highlanders reveals conserved hypoxia tolerance genes
Source: Genome Biol. 2014 Feb 20;15(2):R36. doi: 10.1186/gb-2014-15-2-r36 (PMC4054780; doi:10.1186/gb-2014-15-2-r36)
Supplement: Additional file 16: Table S4 — Sequencing depth and coverage statistics per individual in the sample. [file gb-2014-15-2-r36-S16.pdf]

Table S4. Sequencing depth and coverage statistics per individual in the sample.

Sample	Total Reads	% Mapped	Mean Depth	% Coverage
01E	540,766,668	97.97%	17.17x	99.05%
02E	597,069,058	97.98%	18.93x	99.11%
04E	635,419,642	97.89%	20.15x	99.03%
06E	619,664,584	97.64%	18.86x	99.06%
07E	603,759,184	97.84%	19.08x	99.10%
09E	593,692,762	98.12%	19.01x	99.09%
11E	548,821,162	98.11%	17.61x	99.38%
12E	493,822,992	97.70%	15.76x	99.32%
13E	537,399,998	97.91%	16.77x	99.27%
14E	604,645,336	97.98%	19.01x	99.05%
16E	564,564,486	97.81%	17.79x	99.28%
17E	572,847,150	97.40%	17.69x	99.28%
20E	572,320,746	98.30%	18.27x	99.30%
