# Supplementary material for: Whole genome sequencing of Ethiopian highlanders reveals conserved hypoxia tolerance genes
Source: Genome Biol. 2014 Feb 20;15(2):R36. doi: 10.1186/gb-2014-15-2-r36 (PMC4054780; doi:10.1186/gb-2014-15-2-r36)
Supplement: Additional file 17: Table S5 — Number of variants removed in each filtering step, for the Luhya and Oromos populations. [file gb-2014-15-2-r36-S17.pdf]

Table S5. Number of variants removed in each filtering step, for the Luhya and Oromos populations.

Filtering Step	Oromos	Luhya
GATK Region Filtering (in Luhya)	-	602,188
Satellite DNA filtering	30,684	70,398
Coverage Filtering (in Luhya)	33,222	203,326
