# Supplementary material for: Whole genome sequencing of Ethiopian highlanders reveals conserved hypoxia tolerance genes
Source: Genome Biol. 2014 Feb 20;15(2):R36. doi: 10.1186/gb-2014-15-2-r36 (PMC4054780; doi:10.1186/gb-2014-15-2-r36)
Supplement: Additional file 13: Table S3 — Clinical characteristics of Oromos and Amhara subjects. [file gb-2014-15-2-r36-S13.pdf]

Table S3. Clinical characteristics of Oromos and Amhara subjects.

Sample ID	Age	Native	Blood Pressure	Heart Rate	Respiratory Rate	Saturation PO2	Hematocrit	CMS score															
								Dizziness	Physical weakness	Mental fatigue	Anorexia	Muscle weakness	Joint pain	Breathlessness	Palpitation	Sleep disturbance	Cyanosis of lip, face, or fingers	Injected conjunctive	Dilation of veins in hands/feet	Paresthesia in the hands/feet	Headache	Tinnitus	Total CMS score
01E	37	Oromos	135/80	80	20	90	50	0	0	0	0	0	0	0	0	0	2	0	2	0	0	0	4
02E	38	Oromos	130/90	64	20	89	49	0	0	0	0	0	0	0	0	0	2	0	2	0	0	0	4
04E	32	Oromos	120/80	68	20	89	54	0	0	0	0	0	0	0	0	0	0	2	2	0	3	0	7
06E	32	Oromos	120/85	63	24	84	56	0	0	0	0	0	0	0	0	0	0	0	0	0	3	0	3
07E	33	Oromos	130/80	80	20	88	50	0	0	0	0	0	0	0	0	0	0	2	2	0	0	0	4
09E	30	Oromos	120/90	72	20	87	56.5	1	0	0	0	0	0	0	0	0	0	0	0	0	3	0	4
11E	31	Amhara	135/77	76	20	94	47	0	0	0	0	0	0	0	0	0	0	0	0	0	0	0	0
12E	31	Amhara	130/63	92	24	96	53	0	0	0	0	0	0	0	0	0	0	0	0	0	0	0	0
13E	30	Amhara	131/70	66	22	90	46	0	0	0	0	0	0	0	0	0	0	0	0	0	0	0	0
14E	30	Amhara	134/85	64	22	94	48.5	0	0	0	0	0	0	0	0	0	0	0	0	0	0	0	0
16E	33	Amhara	130/69	71	24	85	49.5	0	0	0	0	0	0	0	0	0	0	0	0	0	0	0	0
17E	34	Amhara	115/71	74	24	87	50	0	0	0	0	0	0	0	0	0	0	0	0	0	0	0	0
20E	32	Amhara	104/61	68	22	89	52.5	0	0	0	0	0	0	0	0	0	0	0	0	0	0	0	0
